# In vitro electrical stimulation devices: practical framework for design, fabrication, and operation

**DOI:** 10.1186/s13036-026-00664-7

**Published:** 2026-03-20

**Authors:** Gaurav Kulkarni, Jorge M. Garcia, Miriam Isasi-Campillo, María Ujué González, Sahba Mobini

**Affiliations:** Instituto de Micro y Nanotecnología, IMN-CNM, CSIC (CEI UAM+CSIC), Isaac Newton 8, Tres Cantos, Madrid, 28760 Spain

**Keywords:** Electrical stimulation device, Electrochemical characterization, Electrical stimulation protocol, In vitro electrical stimulation

## Abstract

**Background:**

Electrical stimulation of cell culture systems has shown significant potential benefits, attracting growing interest in bioelectrical research and tissue engineering. However, implementing effective stimulation remains challenging due to the complexity of device design, protocol selection, and procedure standardization. Many biology-focused laboratories face a knowledge gap in electronics and electrochemistry, complicating the choice of circuits, electrode materials, and configurations, which often leads to a lack of reproducible and standardized protocols.

**Results:**

We present a comprehensive, step-by-step guide for developing a cost-effective, high-throughput, and adaptable in vitro electrical stimulation device containing platinum electrodes. The guide includes a method to develop standard operating protocol, maintenance instructions, and a practical example of device use. We describe an approach to design and approximate electrical stimulation protocols using electrochemical characterization and equivalent circuit modelling. All design files, circuit diagrams, component lists, measurements, and simulations are provided as open-access resources.

**Conclusions:**

This work offers a fully replicable framework for implementing electrical stimulation in any laboratory with basic electronics and electrochemistry knowledge. By providing simulation tools, standardized protocols and accessible design resources, it addresses reproducibility challenges and facilitates broader adoption of bioelectrical stimulation in cell culture research.

**Supplementary Information:**

The online version contains supplementary material available at 10.1186/s13036-026-00664-7.

## Background

Electrical stimulation (ES) of cells and tissues has gained significant attention across various fields of biology and biomedicine over the past decades [1–12]. Although applying electrical signals to cell cultures may appear straightforward, the development of effective devices, the selection of appropriate ES protocols, and the establishment of reproducible procedures remain complex challenges.

ES is produced through multiple approaches, including different delivery types (direct, capacitive, or inductive), modes of stimulation (voltage- or current-controlled), and electrode configurations. All these have been tested in various biological systems and reviewed extensively elsewhere [13, 14]. None of these approaches is exclusively preferred over the others and each has context-dependent advantages. Yet, the focus of this work is on direct ES, where conductive electrodes are directly inserted in the biological environment. Direct ES of cells or living tissues is a common and easy-to-set-up approach. This explains the extensive use of direct ES in clinical settings via implantable electrodes for various applications such as neuromodulation [15, 16], tissue regeneration [17], and healing acceleration [18–20].

Nevertheless, ES outcomes still face persistent challenges in controllability and reproducibility, which largely arise from device and protocol design, standardization and translation of parameters from laboratory settings to clinical devices [21, 22]. Addressing these challenges requires studies at the fundamental level using controllable in vitro platforms and in-depth analysis of their electrical properties and associated electrochemical processes.

While numerous designs for in vitro ES devices have been reported [22–33], researchers still face uncertainty regarding which stimulation protocol or method should be used. This cannot be determined without considering that the stimulation protocol and device are interrelated components; in other words, designing an effective ES protocol cannot be separated from the stimulation device itself. A protocol optimized for a particular platform can only be transferred to another stimulation device if the physical and electrochemical characteristics of both devices are carefully evaluated and the protocol is adapted accordingly. Recently, the electrochemical aspects of bioelectrical stimulation have received considerable attention within the research community [34, 35]. These studies emphasize the electrochemical nature of bioelectrical stimulation, highlighting that directly applying the parameters of a reported protocol (voltage/current, frequency, wave form) to a different setup without proper adaptation is a serious misconception that can result in inconsistent and unreliable outcomes. For example, even under identical geometric conditions and a fixed applied electric field, substantially different current amplitudes and temporal signal evolution are produced inside the culture area simply by changing the electrode material from platinum to titanium [36]. This is because electrode material, surface and geometry determine the electrochemical signature of the system, influencing the charge injection and electric field generation. Therefore, ES protocols and ES devices should be regarded as interdependent components; a standardized, practical framework is needed to facilitate the translation of ES protocols from one device to another.

Our laboratory has a long-standing history of developing ES devices [24, 37, 38]. In this article, we describe a device with significant advancements from the early versions [39]. This device has been extensively tested for reproducibility and is now routinely used in our research. The aim of this article is to openly share a framework for the fabrication of an efficient in vitro ES devices together with practical guidelines to design standardized ES protocols with the broader scientific community, particularly with biology labs who may lack advanced engineering, electronics and electrochemistry expertise.

In this article we described the fabrication of an ES device with parallel-plate Pt nanostructured electrodes on commercial 8-well micro-dishes, providing open-access design files and technical details. This cost-effective approach uses metal evaporation (see cost estimation in the supplementary information) to produce durable electrodes and includes ES protocol design supported by electrochemical characterization and equivalent circuit modelling, with all simulation files and examples provided. In the Results, we show that integrating a measurement circuit enables precise monitoring of delivered electrical parameters, closely matching model predictions, ensuring reproducible protocols across laboratories. Device lifecycle assessments are presented, and the device’s applicability is demonstrated in neural tissue regeneration experiments. Overall, the framework is scalable, adaptable to diverse laboratory settings, and compatible with various 2D and 3D culture platforms, with ready-to-use design files provided.

## Methods

### Electrical stimulation device design and fabrication

**Fig. 1:**
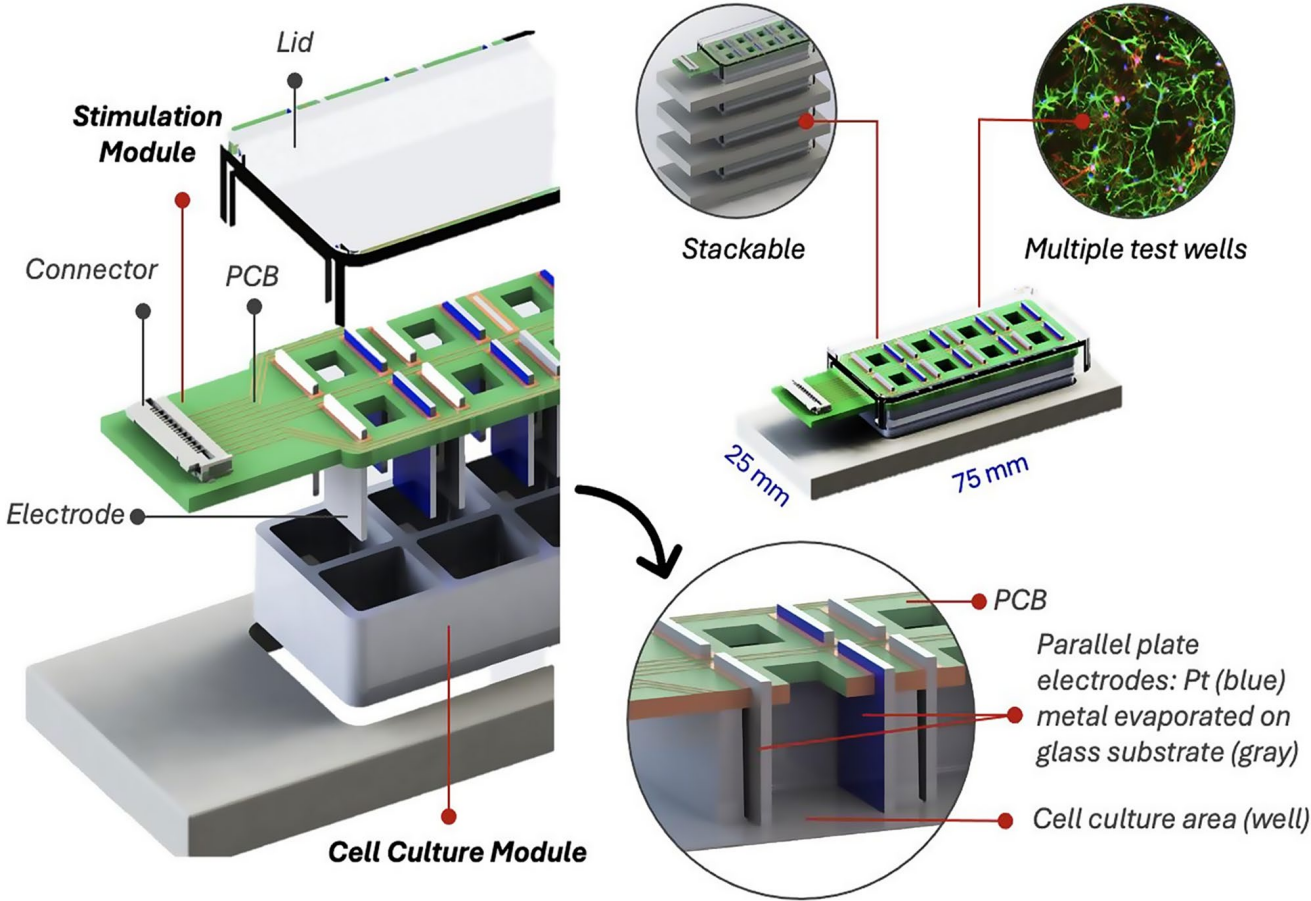
Electrical stimulation device design. Exploded view of the electrical stimulation device adapted for an 8-well micro-dish, highlighting the main components: printed circuit board (PCB) and Pt metal evaporated electrodes (stimulation module), and cell culture module (left). Overall device design with key dimensions, illustrating high-throughput compatibility (top right). The zoomed-in view of a single well, with the stimulation module inserted showing the parallel plate electrode configuration

The ES device developed here incorporates two parallel plate electrodes in each individual culture area and consists of two main compartments: a cell culture module, which maintains the biological sample, and a stimulation module, which integrates the electrodes, electronic circuitry, and connectors, Fig. 1. This design supports reusability, facilitates experimental flexibility, and allows optimization for various ES protocols. Parallel plate configuration was selected over pin or wire configurations due to their ability to generate a uniform electric field across the culture region [40].

We selected an 8-well micro-dish cell culture platform (Sarstedt, Germany) as the cell culture module, Fig. 1. Micro-dish culture platforms are commercially available from several suppliers and offer several advantages. In the parallel configuration the electrode spacing is ~ 10 mm, which permits a field of ~ 130 mV·mm⁻¹ while keeping electrode potentials within the safe electrochemical window for Pt (~ from − 1 V to + 1.3 V). See Section  “Electrochemical basis of direct electrical stimulation” for further explanation on safe electrochemical window. The device footprint is compact (75 × 25 mm), modular, and stackable, enabling high-throughput experimental design (*n* = 8 per platform) as well as convenient handling and storage. A removeable top bracket simplifies downstream processing such as immunofluorescence staining for multiple replicates in one instance. Each well holds ~ 500 µl of culture medium and can accommodate ~ 10,000 to 100,000 cells in monolayer depending on the cell type, which provides a favourable balance between biological throughput and consumable cost. This format is well-suited for reproducible and scalable ES experiments in 2D monolayer and 3D organoid cultures; both have been tested in our laboratory. However, platform choice should always be guided by the experimental requirements and is not limited to the example presented here, see discussion section and supplementary Table S1.

#### Development of electrodes

The selection of electrode materials for bioelectrical stimulation depends on multiple physicochemical parameters, including material electrical conductivity (σ), impedance (Z), electrochemical stability, charge storage capacity (CSC), mechanical robustness, and biocompatibility. Noble metals such as Au, Pt and Pt/Ir alloys remain the most established materials for fabrication of bioelectrodes, offering high conductivity and CSC, low impedance, and excellent corrosion resistance [25, 41–43]. See supplementary Table S2 for ES devices using Pt and Au electrodes. Both Au and Pt are widely used in fabrication of clinically approved stimulation/recording electrodes [44]. Pt is generally preferred over Au because it possesses excellent electrochemical properties due to its unique pseudocapacitive behavior that supports reversible faradaic reactions (e.g., hydrogen adsorption/desorption as well as platinum oxidation) [45, 46], enabling safe and efficient charge transfer with superior CSC [36, 47]. This advantage favors both stimulation and recording applications, where Pt electrodes reduce signal attenuation and increase signal-to-noise ratio more effectively than most clinically approved materials. Besides, Au films are mechanically more fragile. It is worth noting that Pt/IrO_x_ alloys are also highly promising because IrO_x_ provides additional redox-active sites, higher CSC, and improved stability, while Pt ensures efficient electron transport [48]. Yet, the main drawback of noble metals is their high cost and typically being among low abundant materials.

Our practical solution for reducing cost and material is to deposit thin films of Pt on flat surfaces such as polymer or glass by means of metal evaporation techniques. We selected Pt as the electrode material, as it offers excellent properties. It is known that films as thin as 15 nm can reproduce the electrochemical properties of bulk material [49, 50]. Therefore, a ~ 100 nm Pt film guarantees durability and mechanical stability. Furthermore, we [36] and others [47, 48] demonstrated that metallic films can be nanostructured to significantly increase their electrochemically active surface area, resulting in approximately 6-fold enhancement in CSC. Nanostructuration also significantly reduces the cut-off frequency of Pt impedance, enabling stable, low-impedance performance even at frequencies as low as 18 Hz [36]. Nanostructured films can be achieved directly by glancing angle deposition [51], which does not require additional technological step. Although outsourced fabrication of Pt thin films remains relatively expensive, the overall device cost is significantly reduced by eliminating the use of bulk metal (see the detailed cost estimation table in the Supplementary Information).

**Fig. 2:**
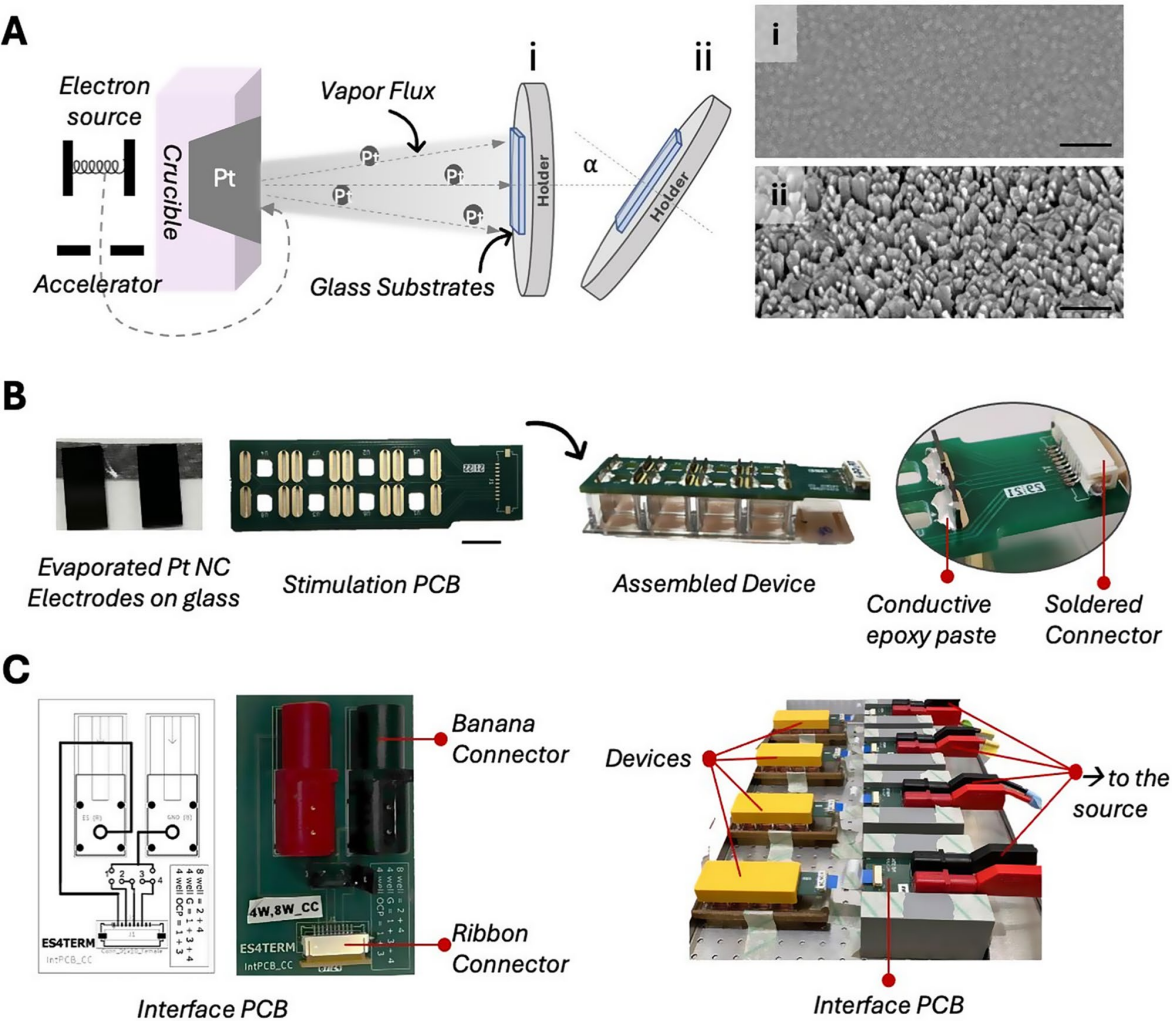
Device fabrication and assembly. **A)** Schematic of Pt electrode fabrication via electron-beam metal evaporation on a glass substrate, either (i) perpendicular to the crucible or (ii) at an angle. Scanning electron microscopy (SEM) top view images of the resulting electrodes (i) thin-film (TF), and (ii) nanocolumnar (NC) Pt electrodes, scale bar = 100 nm. **B)** From left to right, photographs of evaporated Pt nanocolumnar electrodes, the stimulation PCB (scale bar = 10 mm), and the fully assembled device. Zoomed-in view of the electrical connection between the electrodes and the PCB, using conductive epoxy. **C)** Layout and photograph of an interface PCB. This specific interface PCB is configured to connect devices designed for current-controlled stimulation to the signal source while enabling flexible assignment of stimulation and sham/control wells (left). Multiple devices connected to interface PCBs, placed on the incubator tray, and powered by a common source, with both interface PCBs and devices secured using 3D-printed racks (right)

To fabricate Pt electrodes for the 8-well micro-dish, we used 8 pairs of float glass substrates of 7 mm × 14 mm (see Supplementary Table S1 for more substrate dimensions tested and used for other common culture platforms). Float glass was selected as the substrate because it offers a sufficiently smooth, inexpensive surface for reproducible metal deposition, and is universally accepted by evaporation facilities; unlike certain polymers, which may be restricted due to potential risk of introducing contamination in the evaporation chamber. The metal evaporation method and its parameters determine the resulting film thickness and surface roughness. Several techniques are available for Pt deposition, including magnetron sputtering and electron beam evaporation [52, 53]. In this work, we used electron beam metal evaporation, a technique available in standard microfabrication facilities and services, Fig. 2A.

Prior to deposition, pre-cut float glass substrates (Präzisions Glas & Optik GmbH, Germany) were subjected to a sequential cleaning protocol; the substrates were sonicated in acetone and then in isopropanol for 10 min each to remove organic contaminants, followed by rinsing with deionized water and rapid drying using pressurized air or nitrogen. Substrates are loaded in the metal evaporation chamber and 3 nm of Ti are deposited as an adhesive layer. To obtain thin films (TF) the substrate remains parallel to the material crucible, α = 0°; then, 50 nm of Pt is deposited at 0.4 Å/s, and the thickness of the deposited metal is controlled using a quartz crystal microbalance. To obtain nanocolumnar films (NC), the substrate is tilted at α = 80° and Pt is evaporated at the rate of 0.8 Å/s. In this case, due to the increased projected substrate area together with the atomic shadowing effect [53], the calibration of the quartz balance cannot be related to the thickness of the NC film. As a reference, in our system where the distance between the crucible and substrate is ~ 23 cm, a deposition time corresponding to a reading of 300 nm in the quartz balance gives rise to a ~ 200 nm thick NC film. Nevertheless, the thickness of the films can be determined using scanning electron macroscopy (SEM) imaging at cross section. This approach allows the controlled fabrication of both continuous thin films and nanostructured films, thereby tailoring surface morphology to enhance electrochemical performance, Fig. 2A.

#### Printed circuit boards (PCBs) and assembly

The printed circuit boards (PCBs) are designed to securely hold the electrodes in the right position inside the well and to provide electrical connections to the stimulation source, Figs. 2B. For the 8-well micro-dish device, we designed stimulation PCBs with pre-wired circuit layouts to allow multiple wells to be stimulated under identical conditions. Depending on the stimulation mode, voltage- or current-controlled, the wells are wired in parallel or in series, respectively. To ensure high-quality and reliable electrical connections, the vias into which the electrodes are inserted are gold-plated through-holes, minimizing contact resistance and improving signal integrity (See supplementary Table S3, Link S3.1 and S3.2, to download main PCB fabrication files). To assemble the device, we connect the electrodes to the stimulation PCB using conductive epoxy (EPO-TEK H20E, USA), Fig. 2B. An assembly rack (e.g., simply a 3D-printed replica of the cell culture dish) can be useful to hold components in place during this process. According to the manufacturer’s instructions, the epoxy must be cured at minimum 80 °C for 3 h to achieve mechanical robustness. Therefore, assembly rack should be made of temperature operating polymers such as ABS. This step is preferred to be completed before soldering the connectors to the PCB. A list of connectors and cables tested for various platforms is reviewed in supplementary Table S4.

To maintain flexibility in experimental design (e.g., assigning specific wells to stimulation or sham/control groups) an additional circuit module, referred to as the *interface PCB*, is designed, Fig. 2C. The interface PCB enables customizable routing of stimulation signals and facilitates the integration of banana or BNC connectors, by separating these heavier connectors from the stimulation module, improves mechanical stability, and simplifies handling of the miniaturized devices (See supplementary Table S3, Link S3.3.1 and S3.3.2, and S3.4 to download interface PCB fabrication files). Ribbon cable connectors are used to link the stimulation PCBs to the interface PCB due to their lightweight design and versatility. The interface PCB then consolidates signals from multiple wells into a single pair of banana connectors or a single BNC output, allowing easy adaptation to different experimental configurations.

#### Electric signal sources

The choice of signal source is primarily determined by the mode of stimulation, which can be either voltage-controlled or current-controlled.

In voltage-controlled stimulation, the applied voltage is directly regulated by the source. Common voltage sources include USB-DAQ units, function generators, and multi-channel voltage/current stimulators. These devices are typically low-cost and easy to operate. When choosing a source, an important parameter to consider is the maximum frequency. For biostimulation applications, typical frequencies range from a few mHz to several hundred kHz. Thus, high-end GHz sources are unnecessary, reducing overall cost. The ability to design flexible waveforms is also advantageous for exploring different ES protocols.

The maximum output current is another critical factor, as it determines how many wells can be stimulated simultaneously by a single source. In voltage-controlled systems, simultaneous stimulation is achieved by connecting all wells and devices in parallel. Connecting the wells in parallel ensures consistent voltage across each well, despite small local differences in impedance or resistivity. Therefore, the source must provide sufficient current to maintain the nominal voltage across all wells. For instance, with a source capable of delivering 100 mA, only four 8-well devices with a total load of ~ 42 Ω each (~ 330 Ω per well) can be connected in parallel to achieve a delivered voltage of 1 V.

Additionally, the output impedance of the source must be known and considered when designing the ES protocol. Most voltage sources have an output impedance (*Z*_*output*_) of 50 Ω. Consequently, the system acts as a voltage divider and the actual voltage delivered to the wells depends on factors such as the number of connected wells, applied frequency, and device impedance. The use of a monitoring circuit allows us to solve this ambiguity and adapt the nominal voltage value at the source to the desired voltage delivered to the wells before and during the experiment, see Section  “Device in operation”.

In current-controlled stimulation, the source delivers a defined current to the system. In the multi-well devices, the wells are configured in series to ensure uniform net current delivery to all wells. Current generator sources are typically more complex and costly due to their electronic design. Although current sources generally support lower maximum frequencies than voltage sources, this limitation is not restrictive for bio-stimulation. Another drawback is the limited variety of available waveforms; however, this can be addressed by combining a flexible function generator with a current source. Alternatively, multichannel voltage/current stimulators offer versatile and programmable control over multiple parameters. When operating in current-controlled mode, current stability is crucial to ensure safe stimulation, see Section  “Defining relevant ES protocols”. It is also important to consider the maximum output voltage. For example, with an 8-well device (~ 330Ω per well) connected in series, the total load is 2.6 kΩ; therefore, to deliver 3 mA to four devices, an output voltage of ~ 32 V is required.

#### Cleaning, sterilization and repair

Cleaning and sterilization of devices are essential to prevent contamination of cultures and ensure quality and reproducibility of experiments. Prior to each use, electrodes are cleaned by immersion in deionized water and sonication for 3 min, taking care not to submerge the electronic components. Surfaces can then be disinfected by gently spraying with 70% ethanol, followed by rinsing with sterile water under aseptic conditions, and allowing the device to fully air dry before UV exposure. For UV sterilization, devices should be irradiated with dosage of 0.2–2 mW/cm^2^ from multiple angles at least for 20 min in each position. When available, other approved sterilization methods, such as hydrogen peroxide, ethylene oxide, or gamma irradiation, might be applicable to the entire device, though it was not tested directly in our laboratory. We do not recommend oxygen plasma cleaning, since we witnessed that the conductive epoxy was burnt by oxygen plasma compromising the connections. Finally, if an electrode breaks, it can be carefully replaced by removing the old epoxy by means of localized heating with a soldering iron up to 300 °C and then a new electrode would be mounted with fresh epoxy.

### Electrical stimulation protocols design

Designing ES protocols for cells and tissues remains a controversial topic. Many studies adopt parameters from prior reports without fully considering the specific biological context, and reproducibility of the conditions ignoring electrode geometry, material, and culture configurations that determine electrochemical characteristic of the system. These inconsistencies make protocols difficult to reproduce across systems and laboratories and limit the possibility of meaningful meta-analyses [22]. 

The design of an effective ES protocol should first and foremost be guided by the specific biological objective. For example, the biological goal may involve replicating endogenous electrical environments, such as continuous transepithelial potentials or the oscillatory fields found in neuromuscular and cardiac tissues. Alternatively, ES may be employed to enhance therapeutic interventions, including regenerative stimulation, deep brain stimulation, or the modulation of cell migration. In more exploratory contexts, ES parameters can be designed to investigate novel biological responses, such as manipulation of the cell secretome.

Defining the biological goal is critical for establishing meaningful ES conditions. These conditions encompass the magnitude and orientation of electric field delivered inside the culture area (E_e_), the dynamics of the delivered charge (Q), and the temporal characteristics of stimulation events (t). Once these parameters are clearly specified, applied amplitude, waveform and frequency are calculated in accordance with the electrical properties of the stimulation system. Given that biological environments typically involve electrolytic media, the electrical behaviour of the system is governed by electrochemical interactions at the electrode–electrolyte interface. Therefore, beside the biological aim, it is essential to incorporate electrochemical design principles to ensure safe, effective, and reproducible ES protocol.

#### Electrochemical basis of direct electrical stimulation

Direct ES of cells and tissues inherently involves electrochemical phenomena occurring at the electrode–electrolyte interface, where the extracellular fluid acts as the electrolyte [34, 45, 54]. The electrode–electrolyte systems cannot be described simply by Ohm’s law (*V = I* × *R*) because the interface is not purely resistive. At the interface an ionic double-layer is formed, which acts as capacitor and makes the electrical behaviour time- and frequency-dependent [55]. Therefore, we use impedance (*Z*), which combines reactance and resistance, to describe voltage–current behaviour.

During ES, when electrodes are placed inside the cell culture area, an electric source injects electrons to the cathode, charging it negatively, or collects them from the anode generating a positively charged electrode. At the electrode–electrolyte interface, the charged electrode surface induces a redistribution of water molecules and ions, forming an electric double layer [45]. The electric double layer behaves as a capacitor with a capacitance value, C_dl_, and its associated capacitive reactance, $\:{Z}=\frac{1}{{j}\times 2\pi \times{f}\times{C}_{dl}}$, where f is frequency, and j is the imaginary unit, which represents the 90° phase shift between voltage and current in a capacitor. The value of C_dl_ strongly depends on the intrinsic properties of the electrode material (e.g., conductivity, porosity, roughness) as well as its effective surface area. Environmental conditions, including temperature and electrolyte composition, further modulate C_dl_. Thus, determining C_dl_ under relevant experimental conditions is essential for accurately predicting the delivered stimulation waveform. When C_dl_ is a large value, the charge and discharge of the double layer is slow and therefore, the waveform and the amplitude of the applied signal are better preserved.

Inside the electrolyte, the movement of the ions (e.g., chloride (Cl⁻), phosphate (PO₄³⁻), sodium (Na⁺), and potassium (K⁺)) under the applied electric field gives rise to an ionic current. From an electrical perspective, the bulk electrolyte can then be approximated as a resistor, denoted Rₑ. The value of Rₑ highly depends on the composition, temperature, concentration and the distance between the electrodes (in parallel configuration).

Moreover, electrons with sufficient energy at the interface cause electrochemical reactions and participate in reduction or oxidation, collectively referred to as faradaic reactions. These processes are represented by a charge transfer resistance, R_f_. The value of R_f_ is dynamic and closely linked to the required energy of faradaic reactions, mainly associated with electrode material and effective surface area as well as stimulation parameters. Most faradaic reactions are irreversible [45]. One of the well-studied examples of faradaic reactions in bioelectrical stimulation is the generation of reactive oxygen species (ROS) that diffuse to the electrolyte. ROS become increasingly interesting in this context since it can largely modulate biological responses in a concentration-dependent manner [56–58]. However, certain electrode materials such as Pt can support surface-confined (not diffused to the bulk), reversible redox reactions that contribute to pseudocapacitive charge injection within a limited electrochemical window.

**Fig. 3:**
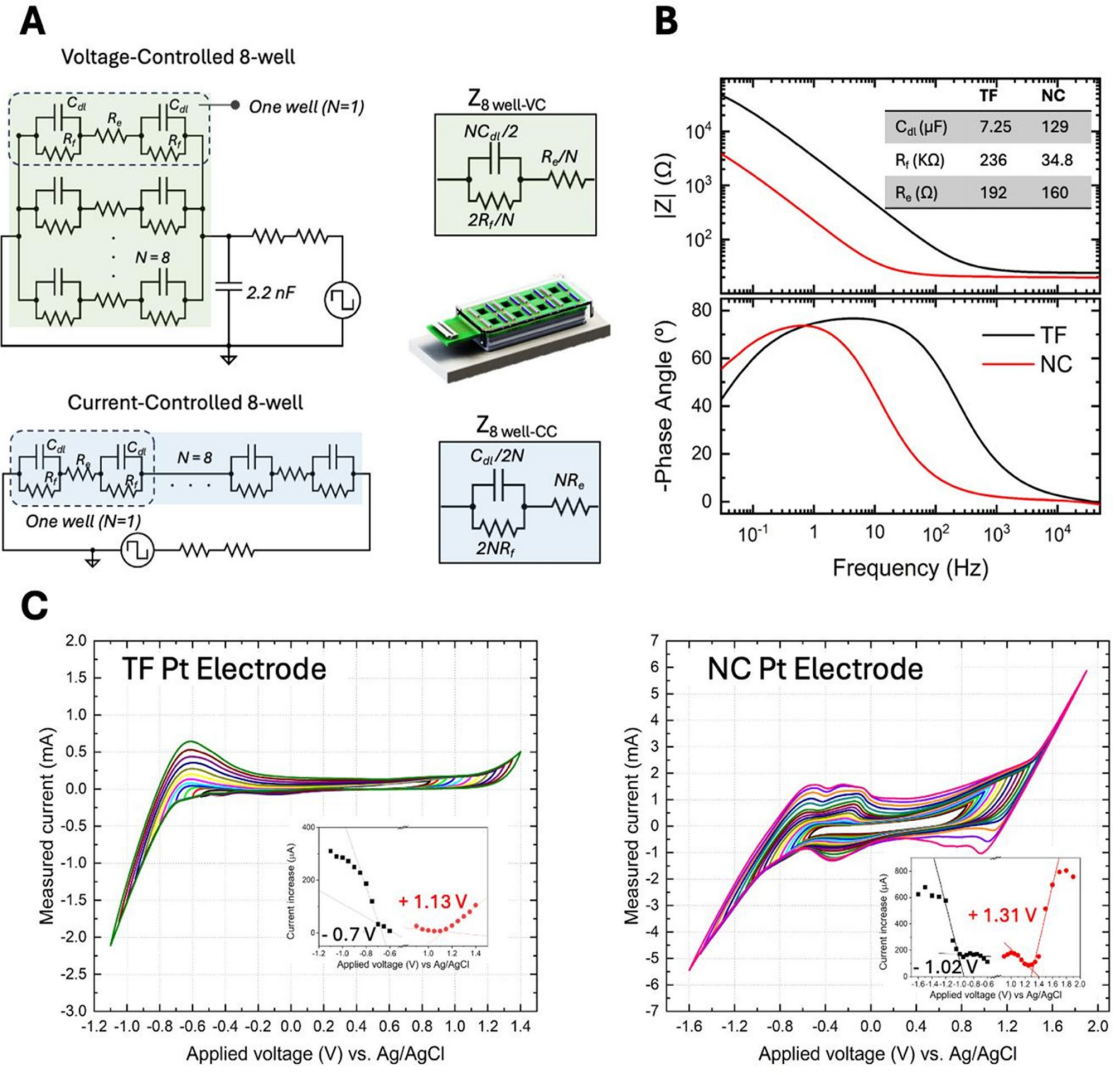
Equivalent circuits and electrochemical characterizations. **(A)** Equivalent circuits for a single well and 8-well micro-dish under voltage-controlled and current-controlled stimulation modes (left). Simplified versions of equivalent circuits for all wells connected: Z_8 well-VC_ and Z_8 well-CC_ (right). **(B)** Bode plots from EIS measurements of the 8-well micro-dish in voltage-controlled mode, comparing thin film (TF) and nanocolumnar (NC) Pt electrodes under standard culture conditions (37 °C, 5% CO₂) with DMEM as the electrolyte. The inset table shows the average values of C_dl_, R_f_, and R_e_ obtained from several EIS measurements. **(C)** Current-voltage curves obtained from consecutive CV measurements in a single well with DMEM as the electrolyte, under standard culture conditions (37 °C, 5% CO₂). Insets show current increase vs. voltage plots, used to estimate safe voltage range

Overall, a typical direct stimulation setup translates to an electric circuit with C_dl_ and R_f_ connected in parallel, representing the electrode-electrolyte interface, both connected in series with R_e_, which accounts for the bulk resistance of the electrolyte [34, 45]. For devices with parallel electrode configuration, this equivalent circuit provides a simplified yet informative framework to analyse charge transfer dynamics and optimize stimulation parameters. Figure 3A shows the equivalent circuit for single and multiple wells (e.g., N = 8). The overall configuration depends on the stimulation mode: in voltage-controlled systems the wells are wired in parallel, whereas in current-controlled systems they are wired in series. The simplified circuit for each stimulation mode describing the full device as a combination of a single capacitor, a faradaic resistor and an electrolyte resistor is also included in Fig. 3A. The relationship between these simplified values and those of the single well, denoted as Z_8 well-VC_ and Z_8 well-CC_, are also indicated in the sketch. Equivalent circuits are powerful tools for predicting the behaviour of ES systems under given ES parameters. Values of equivalent circuit elements can be experimentally determined using electrochemical impedance spectroscopy (EIS) using a potentiostat. We used a potentiostat from Autolab GSTAT204 with FRA32M module for running the measurements in the 8-well micro-dish device with NC and TF Pt electrodes. Briefly, all wells were filled with culture medium, specifically serum-free, glucose- and pyruvate-free DMEM (Life Technologies, Gibco), and maintained in standard culture conditions (37°C, 5% CO₂). All working electrodes were connected in parallel interfacing with the working and counter terminals of the potentiostat. EIS was conducted in 2-electrode configuration over a frequency range of 10 mHz to 100 kHz using a sinusoidal signal with an amplitude of 10 mV. EIS measurement provides the real (Z’) and imaginary part of impedance (Z’’), which can be plotted as Bode and Nyquist plots. EIS data (Bode plots) in voltage-controlled configuration are shown in Fig. 3B. We used NOVA 2.1 software (alternative software such as Z-view are also applicable) to simulate and obtain the values of the equivalent circuit elements from these data. More details on EIS and equivalent circuits are discussed comprehensively elsewhere [59, 60]. For more accurate description of electrochemical behaviour, the capacitive component of the interface is simulated by so-called constant phase element (CPE). C_dl_ value is directly calculated from CPE using formula described previously [36, 59]. The values of equivalent circuit elements obtained for devices with NC and TF Pt electrodes are shown in Fig. 3B. Once the circuit parameters are determined, a simulation tool can be created using open-access software such as LTSpice, CircuitJS, or QUCS to explore various stimulation parameters (e.g., frequency, amplitude, and waveform) to study and calculate the temporal evolution of Q and E_e_. For the labs with limited access to electrochemical characterization, we provide the LTSpice files for modelling 8-well micro-dish stimulation devices, as a guide to generate their own models or to use as a tool to understand the influence of different ES parameters, supplementary Table S5.

Another relevant aspect to be considered for the direct ES is the voltage values where the electrochemical reactions at the interface of electrode-electrolyte take place. It is critical to determine the safe amplitude limits for stimulation. The maximum amplitude for stimulation of biological systems is typically restricted by the onset of undesired electrochemical reactions, which can lead to the release of harmful chemical species for biological entities. In aqueous environments such as cell culture media, applying excessive voltage to the electrode ultimately triggers the electrolysis of water, resulting in gas evolution and drastic pH changes that can cause severe cellular damage or immediate cell death. Therefore, identifying the safe voltage range, where only non-damaging electrochemical reactions occur, is essential. Cyclic Voltammetry (CV), where voltage is repetitively scanned within a range and generated current is recorded, serves as a valuable tool for probing the presence of electrochemical reactions at different voltage levels. These redox reactions appear as peaks in the current-voltage diagram [61]. 

To determine the safe voltage range for our devices, CV was performed in a three-electrode configuration using an Ag/AgCl (RE-1B, BioLogic) as a reference electrode. Importantly, although the reference electrode is not present during ES experiment, from a safety perspective, defining the potential window based on 3-electrode system CV ensures that faradaic processes are not underestimated, which is particularly relevant for long-term or repeated stimulation protocols. Measurements were conducted in a single well containing DMEM as the electrolyte, under standard culture conditions (37 °C, 5% CO₂), with a sweep rate of 0.1 V/s and a step size of − 1 mV. We started at [-0.55 V to + 0.85 V] to stand below the generic reported values for water splitting with Pt electrode [62]. Consecutive cycles were recorded while progressively expanding the potential window by ± 0.5 V increments until the increase of current at the limits of the voltage window is significant. The absolute value of the difference between the current readings at both voltage extremes for consecutive cycles are plotted, Fig. 3C. A pronounced increase in current at specific voltages indicates the activation of a new charge injection mechanism, in this case water splitting. If the voltage window is extended beyond these voltage values, the water splitting will become visible, and bubbles appear at the electrodes. Results show that for Pt TF electrodes the safe voltage range is [-0.7 V to + 1.1 V], while for NC Pt in extends to [-1 V to 1.3 V].

#### Defining relevant ES protocols

Once the biological objective is established and the electrochemical characteristics of the ES system are assessed, the ES protocol can be designed. A systematic strategy should be used to obtain the desired magnitude and orientation of E and Q as well as their temporal evolution. Here, we outline the main criteria to consider:

##### Choice of ES mode: voltage vs. current

While voltage and current are interdependent, voltage driving current and current generating voltage, the system behaves differently depending on which parameter is actively controlled. Voltage-controlled stimulation offers several advantages that make it a practical and widely adopted approach. Its implementation is straightforward, and the required equipment is relatively inexpensive. Moreover, voltage-controlled stimulation allows the applied voltage to be fixed within the safe voltage range thereby reducing the risk of electrolysis and harsh faradaic by-product formation. The waveform design is highly flexible, allowing for monophasic, biphasic, and asymmetric stimulation. Despite these benefits, voltage-controlled mode has notable limitations. The temporal evolution of the delivered current does not mirror that of the applied voltage and is strongly influenced by the system impedance, *Z*. As mentioned before, *Z* depends on electrode material and geometry and electrolyte composition. As a result, the delivered current for the same applied voltage varies across different systems or experimental setups. Moreover, in voltage-controlled stimulation, the delivered current decays as the double layer forms, see supplementary Figure S6. Current decay leads to the reduction of electric field inside the culture area, so that may reduce the effectiveness of steady-state or long pulse stimulation.

By contrast, current-controlled stimulation delivers a fixed amount of current, which produce consistent electric field regardless of changes in *Z*. However, this benefit come at the cost of greater circuit complexity and stricter bio-safety considerations. To keep the current constant, the voltage at the electrode is continuously increasing as the associated double layer capacitor accumulates more charges, see supplementary Figure S6. Since current-controlled systems do not allow the spontaneous discharge, biphasic stimulation is typically required to remove the charge stored at the electrode interface. However, even small mismatches between the delivered and recovered charge can lead to voltage build-up that eventually exceeds the safe voltage range, posing risks of electrode corrosion, system failure, or cellular damage. Therefore, charge-balanced biphasic waveforms need to be designed to prevent this voltage build-up. The choice of waveform is then more limited in current-controlled mode than in voltage-controlled mode. Moreover, the voltage value at the electrode achieved at every pulse should be kept inside the safe voltage range to prevent harsh electrochemical reactions.

In both voltage and current-controlled modes, continuous monitoring of the delivered signals is beneficial, see Section  “Device in operation”. In the case of current-controlled systems, the monitoring is even essential to detect deviations from the safe voltage range; besides, additional feedback circuit may be used to compensate the mismatch between delivered and recovered charge. All these can be tested using the equivalent circuit simulation prior to experiments.

In summary, voltage-controlled stimulation is advantageous when strict polarization constraints are required to prevent exceeding known redox potentials, thereby minimizing ROS generation or any detrimental reaction and enabling controlled investigation of electrochemical mechanisms. Current-controlled stimulation, in contrast, is preferable when precise charge delivery is essential, as in neural activation, where the biological response is determined primarily by the injected charge rather than electrode potential. Importantly, neither current- nor voltage-controlled stimulation is inherently superior: the optimal mode is dictated by whether the principal consideration is electrochemical safety (voltage limits) or biological efficacy (charge delivery).

##### Waveform, amplitude and temporal parameters

The choice of waveform critically influences both the safety and effectiveness of ES and strongly depends on the mode of stimulation. Continuous stimulation (f = 0 Hz), which in many reports is referred to as direct current (DC) stimulation, is commonly suggested to mimic endogenous bioelectric field. Voltage DC stimulation is relatively straightforward to implement. However, saturation of the double layer significantly reduces the current density through the bulk solution over time, decreasing the electric field significantly [57]. Therefore, the driving force for the biological responses reported in DC stimulation remains ambiguous; it could be the residual electric field associated with faradaic current, the presence of ionic gradients, or chemical reactions at the electrode-electrolyte interface. Indeed, electrodes during DC stimulation are at increased risk of corrosion, generating faradaic by-products. To separate the effect of electric field and oxidative by-products, ROS scavengers such as sodium pyruvate are reportedly used [58, 63]. A practical alternative to DC is to use monophasic periodic pulses (f ≠ 0 Hz) with/without offset in voltage-controlled mode, which emulate DC-like stimulation but periodically restoring the electric field. Biphasic pulses, symmetric or asymmetric, are also relevant. In the case of current-controlled stimulation, charge-balanced signals are mandatory as discussed above (see supplementary Figure S6). The stimulation amplitude is fundamentally constrained by keeping the voltage in the safe range, which is discussed in Section “Electrochemical basis of direct electrical stimulation”. However, the optimal amplitude and waveform for each biological system remain subjects of ongoing research.

Defining temporal parameters of ES protocol remains active areas of investigation. Evidence suggests that the temporal components of the ES signal can play a key role in modulating distinct cellular responses; [64, 65] yet, definitive one-to-one relationship between specific frequencies and biological outcomes have not been verified. Therefore, these parameters should be designed to serve either a native physiological objective (e.g., mimicking endogenous frequencies of heart) or a therapeutic goal with exploratory approach [66, 67]. The temporal parameters of electrical stimulation, frequency and pulse width, are interrelated. The choice of stimulation frequency is primarily dictated by the bioelectrical application. The temporal behaviour of ES systems is governed by the time constant, τ = R_e_ ⋅ C_dl_, an intrinsic property of the electrode–electrolyte interface. τ determines the temporal evolution of the voltage and current response (see supplementary Figure S6). Ideally the shape and amplitude of the delivered signal are closer to those of the applied signal when the pulse width is smaller than τ. Moreover, with the pulse width is much larger than τ, in voltage-controlled ES the delivered current suffers strong decay (limiting efficiency), and in current-controlled ES there will be a large voltage increase (limiting safety). ES devices with larger τ are more flexible for designing broader range of ES protocols, mainly for low frequency applications. τ can be increased through careful electrode design. For instance, electrodes with higher C_dl_ (e.g., NC Pt introduced in this work) exhibit slower current decay and a lower frequency cut-off [36].

The simulation tool introduced in this article help researchers to evaluate the electrical behaviour of the system under specific stimulation parameters before experimenting with biological samples. Simulation of the ES protocol using the equivalent circuit model provides a sufficiently accurate approximation of the signal delivered to cells over time.

## Results

### Device in operation

Once the devices are prepared and the protocol is designed, the setup is ready to operate. Depending on the characteristics of the devices and the ES protocol, a source with adequate output specifications is needed. Several devices can be connected to the same source, enhancing experimental efficiency and throughput, considering the source specifications (Section  “Electric signal sources”). To monitor and control the signal delivered to each well without relying on theoretical calculations, a monitoring circuit can be used.

Loose connections can be a major source of errors and irreproducibility. To ensure stability, all circuits should be secured and fixed; custom-built 3D-printed racks are a convenient solution, see ready-to-print examples in supplementary Table S7. Enclosing the circuits in boxes may help to reduce electronic interferences. Devices and interface PCBs are placed inside the incubator during the experiment; to ensure comfortable handling, the use of stainless-steel tray is recommendable. ES devices or interface PCBs are connected to the source or monitoring circuit using cables with sufficient length to extend outside the incubator once it is closed. Flexible banana cables are well-suited for this purpose. BNC cables are used to link the monitoring circuit to the signal source and oscilloscope, minimizing noise and preserving signal fidelity. Supplementary Figure S8 illustrates multiple devices in operation, their connection configurations, and includes a short video demonstrating all operational aspects.

**Fig. 4:**
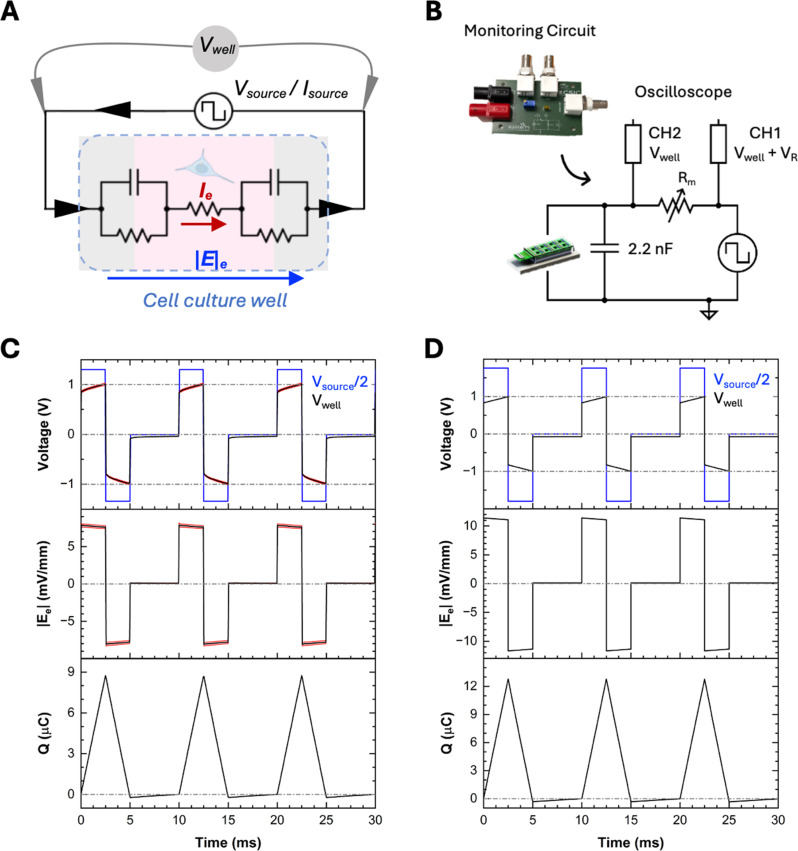
Delivered signal in experiments and simulations. **(A)** Equivalent circuit indicating V_source_ / I_source_, V_well_, I_e_, and |E_e_|. **(B)** Monitoring circuit and its relevant connections to the probe, CH1 and CH2. **C)**. Average values of V_source_, V_well_, Q, and |E_e_|, for 3 days experiment, acquired from monitoring circuit, while two 8-well devices were connected to the voltage source in parallel. Standard deviation plotted in red, demonstrates very small error. **D)** Simulated data acquired from equivalent circuit in the same conditions

Considering the electrochemical impedance of ES devices and the characteristics of the signal sources, the input parameters set at the signal source (V_source_ or I_Source_) are not necessarily the same as those delivered to the cells (V_e_ and I_e_), Fig. 4A. This ambiguity is a major source of confusion in many reports and often contributes to poor reproducibility across laboratories. The electrochemical properties of the system, described in Section  “Electrochemical basis of direct electrical stimulation”, determine the signal amplitude attenuation and waveform modification of the delivered electrical parameters (Q and E_e_) inside the culture area. The delivered signal is also influenced by *Z*_*output*_ of the source, as explained in Section  “Electric signal sources”. To reproduce experiments across several settings and laboratories, reporting the delivered parameters (V_e_ and I_e_) or at least the equivalent circuit of the full system is crucial. This will also help translation of ES protocols from in vitro to in vivo experiments. To address this challenge, adding a monitoring circuit in the setup is a practical solution. Continuous monitoring not only allows for the calculation of charge injection and the electric field applied during stimulation, but also facilitates the detection of unexpected events, which is critical for reliable data analysis and quality control.

We designed a monitoring system to provide on-site real-time measurements of the signals delivered at the ES device, see Fig. 4B, and the supplementary Table S3, Link S3.11 for the PCB. In Fig. 4B we define different electric parameters of an ES system with a single well. V_well_ represents the voltage applied to the well, while V_e_ is the resulting voltage within the cell culture area considering the building of the double layer. The net circulating current through the full circuit including the cell culture area denoted as I_e_. V_well_ determines if we are inside the safe voltage range, see Section  “Electrochemical basis of direct electrical stimulation”, and V_e_ allows to obtain the electric field inside the culture area, |E_e_| = V_e_ / d. Q is calculated by the time integral of I_e_. The monitoring circuit can be used in both voltage-controlled and current-controlled modes. The circuit contains a preset resistor that is set usually at R_m_ = 50 Ω. The voltage across the resistor, V_R_ = V_CH2_ - V_CH1_, allows us to obtain the instantaneous current of the system, which corresponds to I_e_, V_R_ / R_m_ = I_e_. Therefore, |E_e_| can be calculated from V_e_=I_e_⋅R_e_, where R_e_ is determined from EIS measurement, see Section  “Electrochemical basis of direct electrical stimulation”. V_well_ can be directly accessed (V_CH2_). In case of multiple wells (N wells), in voltage-controlled system, the current circulating through R_m_ corresponds to the total current of the system. Assuming all wells are identical, the I_e_ for each well is = V_R_ / (R_m_ ⋅ N). V_well_ is equal for all wells as they are connected in parallel. In current-controlled systems, the current circulating through R_m_ coincides with I_e_ as they are connected in series. V_CH2_ corresponds to the total voltage drop across all wells. For each well, V_well_ = V_CH2_ / N. We use an individual monitoring circuit per device. To avoid loose connections and provide an easy-to-work platform for multiple devices, a 3D-printed rack is designed to immobilize the circuits outside the incubator, see supplementary Table S7.

Figure 4C shows an example of this monitoring circuit operating in voltage-controlled mode, using an 8-well micro-dish NC Pt electrode device under biphasic stimulation with break at 1 V, 100 Hz and a 50% duty cycle (duty cycle= pulse width ⋅ frequency ⋅ 100%). Data were collected twice for each experiment, in the beginning and end of stimulation, over three consecutive days of stimulation. The standard deviation (shown in red) indicates minimal error and fluctuation, confirming the reproducibility of the experimental conditions across different days and moments.

Results show that there is a difference between the V_source_ and V_well_ due to the impedance of the sources and system as explained before. Thus, what needs to be adjusted, keep constant and reported is V_well_ (the voltage arrives at the electrodes). Q and |E_e_| are plotted using the values acquired for I_e_ and R_e_, as described before.

The same parameters were obtained through simulation using the equivalent circuit model (Table S5, Link S5.1), Fig. 4D. The simulated values and waveforms of V_well_ as well as the trends of Q and |E_e_| closely resemble the experimental results. However, the absolute values of Q and |E_e_| are slightly higher in the simulations compared to experiment. This inconsistency arises because the characteristics of the entire system cannot be fully reproduced by the model, although the agreement remains close. Therefore, incorporating a monitoring circuit is highly valuable for acquiring accurate experimental data. Nonetheless, the model provides a sufficiently reliable basis for the design and optimization of the ES protocol, as it correctly predicts the trends of the parameters.

Overall, the monitoring circuit, as a compact add-on to the system, remarkably benefits the standard operation and control of ES experiments. It also provides acquisition of a useful and convertible set of parameters that guarantees the reproducibility of such experiments.

#### Device life cycle

**Fig. 5:**
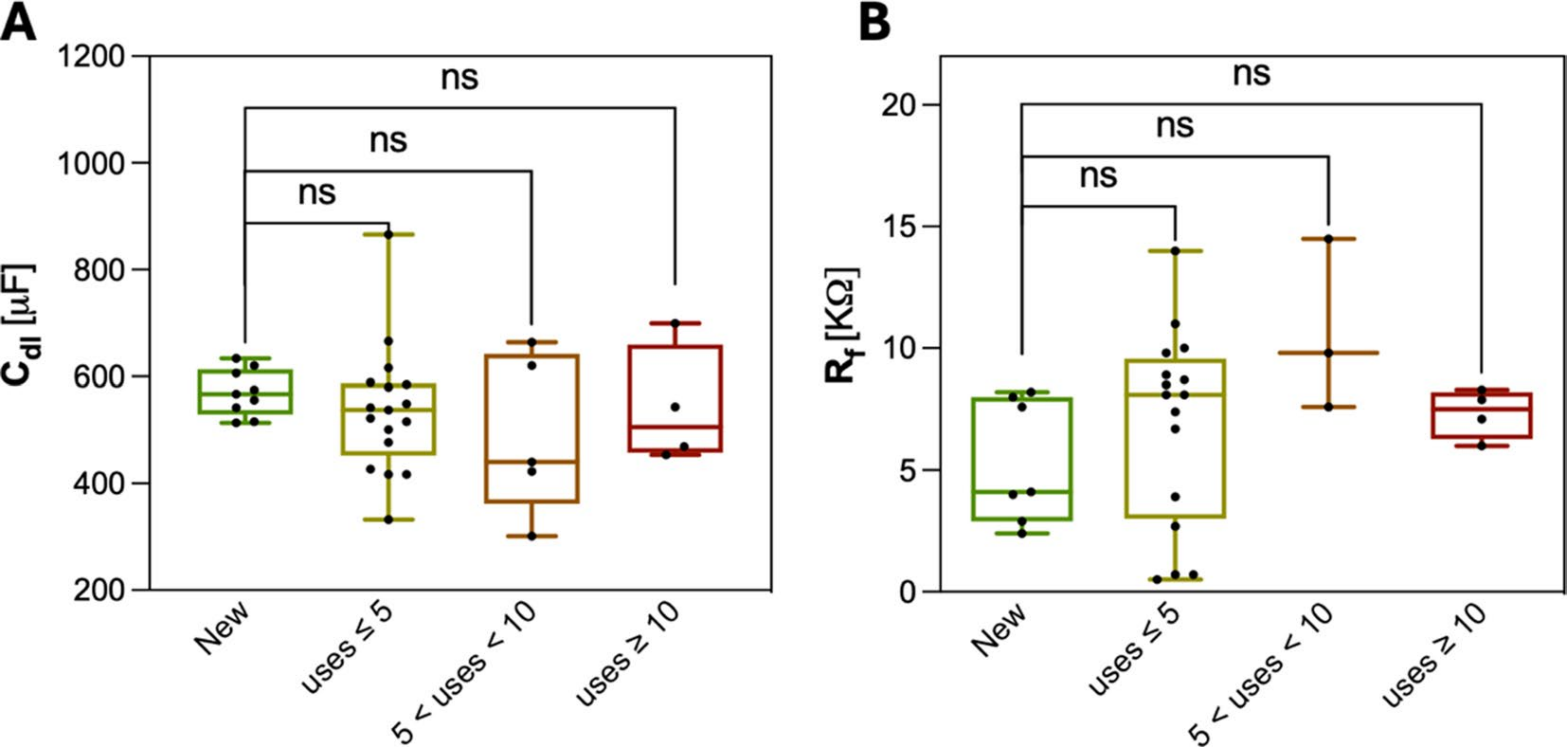
Device life cycle. **A)** C_dl_ and **B)** R_f_ values were obtained from EIS measurements on a minimum of n = 3 and a maximum of n = 8 devices. Each point represents an independent measurement of the same or similar device and is plotted as a function of the number of uses. The average values for both C_dl_ and R_f_ show no significant changes after ten or more uses, indicating that the devices maintain their characteristics over multiple experiments and during the time. One-way ANOVA was used to compare all groups with the ‘New’ devices (with no use). No statistically significant differences were observed (*p* > 0.05 in all cases; ns = not significant)

The nature of the electrodes presented in this work, nanometric metal films evaporated onto glass, raises the question on durability of the electrodes and how many times devices can be reused with similar quality. Previously, we evaluated the aging of these electrodes using a reactive accelerated aging test, simulating one year of harsh in vivo conditions by exposing them to 10–20 mM hydrogen peroxide at 85 °C for ~ 13 days [36]. The results indicated that these thin films are highly robust and can withstand such conditions while maintaining their electrochemical properties within acceptable limits. In this work, we evaluated the device durability and performance in vitro, by measuring their impedance using EIS after certain number of uses. The stability of impedance over time confirms that the equivalent circuit parameters remain constant, preserving electrochemical conditions equivalent to those of a newly fabricated device. As shown in Fig. 5, the C_dl_ and R_f_ values of the 8-well micro-dish NC Pt electrode devices were evaluated before and after multiple uses, and the results reveal no significant changes with repeated operation. The statistical non-significant changes were further confirmed by applying every measured C_dl_ and R_f_ to the equivalent circuit and simulating I_e_ and V_well_ using the same ES protocol.

We recommend this quality-control EIS measurement to monitor overall quality of devices. When impedance deviate significantly from the average, connections and electrodes should be inspected and replaced if necessary. When EIS measurement is not accessible, the values collected from the monitoring circuit can give a hint on the healthy performance of the device. Unexpected changes in I_e_ and V_well_ for known ES conditions indicate degradation of the electrodes or the connection.

### Proof-of-concept experiment

The devices and procedures described here are routinely used in our laboratory to investigate the effects of ES on tissue regeneration, particularly in neural recovery following ischemic stroke. We present a proof-of-concept experiment using an 8-well micro-dish device with NC Pt electrodes to apply our standardized procedure in a biological context. The aim was to evaluate whether a low-intensity, low-voltage ES protocol can reduce apoptosis and promote neuronal recovery after injury. Specifically, we examined neuronal responses to excitotoxic injury, a hallmark of stroke, under anodic ES (100 mV.mm^− 1^, 100 Hz, 25% duty cycle). The effects of stimulation parameters such as mode, amplitude, waveform, frequency, and pulse width remain under investigation.

Based on the framework discussed in this article, we justify an ES protocol that can serve as a rational starting point: voltage-controlled stimulation mode was chosen as it is straightforward to implement, and it allows the application of anodic pulses. The E of 100 mV.mm^− 1^ was determined by the maximum electric field achievable using this device (d = 10 mm) while V_well_ is kept within the safe voltage range of Pt NC electrodes (~ -1 V to + 1.3 V). As τ for this device is 2.5 ms, 100 Hz and 25% duty cycle (2.5 ms pulse width) provide a reasonable charge injection with moderate decay of |E_e_|.

**Fig. 6:**
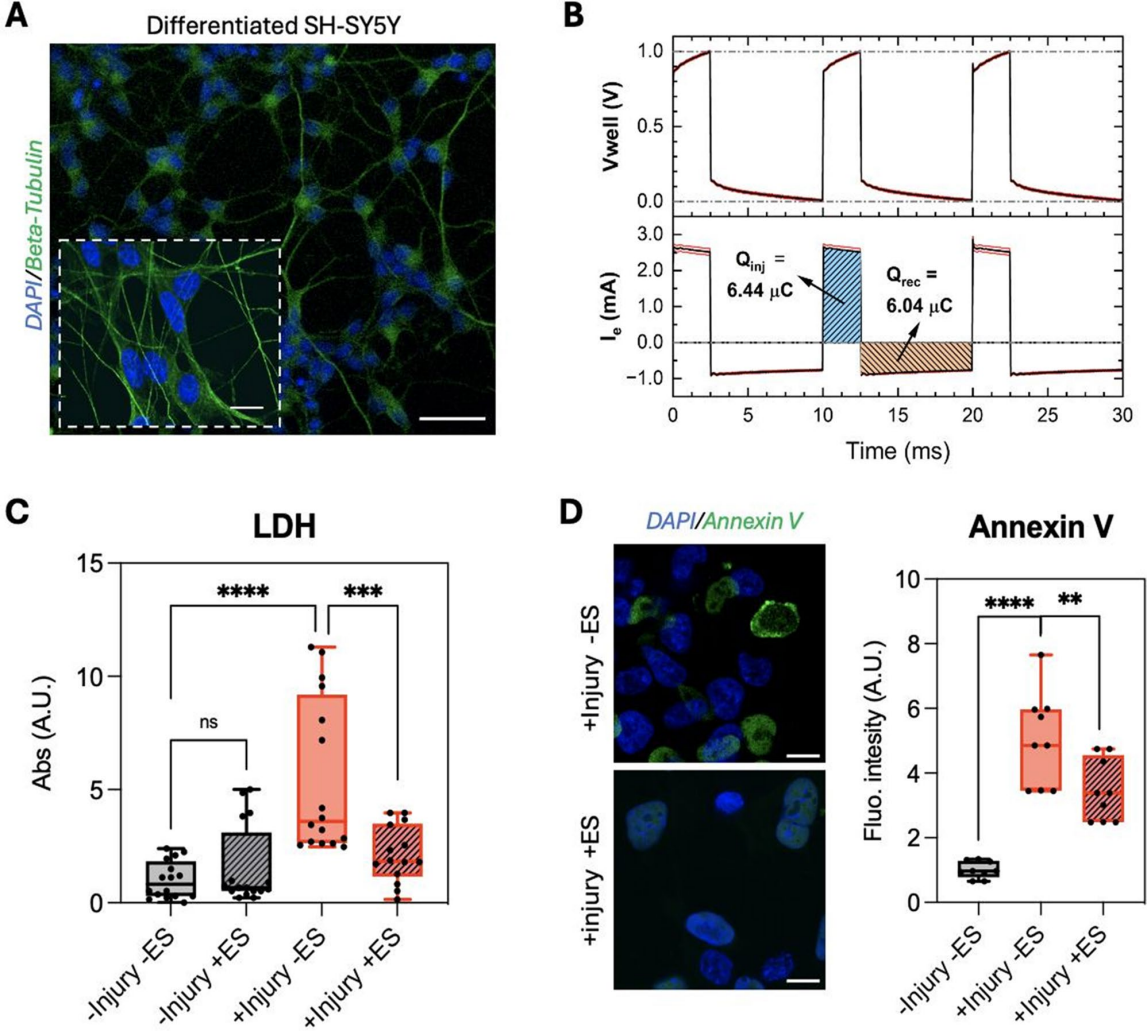
Effect of anodic pulses on neuronal survival following excitotoxic damage. **(A)** SH-SY5Y cells cultured in 8-well micro-dish and differentiated into neuronal-like cells with extended neurites. Cells were stained with DAPI (blue) and anti–β-tubulin (green). Scale bar = 50 μm; zoom-out scale = 10 μm. **(B)** Voltage-controlled anodic pulses (100 mV.mm^− 1^, 100 Hz frequency, 50% duty cycle) were applied for 1 h. Graphs show the acquired data using monitoring circuit, V_source_, V_well_, and the delivered current and charge, measured and calculated according to the standard procedures described in this article. **(C)** Lactate dehydrogenase (LDH) assay showing the proportion of dead cells under various experimental conditions. **(D)** Fluorescence microscopy images of cells stained with Annexin V (green) and DAPI (blue). Scale bar = 10 μm (right). Quantified data (left) represent fluorescence intensity measurements for several wells and images. Data show mean ± S.D. Statistical analyses were performed using ordinary one-way ANOVA; *p* > 0.05 ns = not significant; **p < 0.01; ***p < 0.001; ****p < 0.0001

To create the biological model, human neuroblastoma SH-SY5Y cells were seeded at a density of 10,000 cells/cm² in growth medium (DMEM supplemented with 10% FBS and 1% antibiotics) and maintained in a humidified incubator at 37 °C with 5% CO₂ for 24 h. Cells were then washed with warm PBS to remove excess FBS and exposed to pre-differentiation medium (Neurobasal + B27 + 10 µM retinoic acid + GlutaMAX) for 5–6 days, with medium changes every 2–3 days. Subsequently, cells were treated with brain-derived neurotrophic factor (BDNF) at concentration of 50 ng/ml for 3 days. Figure 6A shows differentiated neuronal cells in an 8-well micro-dish platform and stained with anti-β-tubulin, exhibiting typical neuronal morphology characterized by extensive neurite branching and ramification. Next, cells were subjected to 60 mM glutamate for 60 min, to induce excitotoxic injury. This intervention resulted in ~ 25% cell death and induced apoptotic cells at different stages, together with healthy cells. On the next day, injured cultures were treated with the ES protocol previously described, for 1 h per day over 3 consecutive days. To apply ES, we used a signal generator (Agilent 33220 A); V_source_ was adjusted until the maximum value of the V_well_ pulses was 1 V, measured by oscilloscope (Rohde & Schwarz RTB2004) through the monitoring circuit. Figure 6B shows the average recorded values of V_well_ and I_e_. The integral of I_e_ during the time of applied pulse corresponds to the charge injected in the system, Q_inj_. Integration from the end of the pulse to the start of the next defines the recovered charge, Q_rec_. We should note that during this period of time I_e_ is negative as it reflects the discharge of the system and degradation of the double layer. The difference between Q_inj_ and Q_rec_ provides the charge accumulated during one pulse of ES. This may serve as an interesting discussion point, linking the charge dynamics arising from different ES modes and parameters to the resulting biophysical changes of the system.

One day after the final stimulation, lactate dehydrogenase (LDH) release was measured as an indicator of cell death to evaluate whether ES reduced apoptosis-induced cytotoxicity, Fig. 6C. Importantly, ES had negligible effects on non-injured cells (-Injury + ES), shows that the applied stimulation regime did not damage healthy cells. Cells were then stained with DAPI and Annexin V antibody to identify apoptotic cells. The results showed a significant improvement in decreasing the number of Annexin V positive cells normalized by cell number, indicating cell survival is elevated in the ES-treated group compared to injured, non-stimulated controls, Fig. 6D. This result supports the potential protective effect of ES on cells underwent excitotoxic injury.

These preliminary findings, obtained by high throughput rendering, demonstrate that low-intensity ES delivered by the miniaturized Pt NC electrode device has considerable effects on neuronal cell survival. To further evaluate the potential of ES as a therapeutic strategy for mitigating neuronal loss in stroke and related neurodegenerative conditions, additional stimulation parameters need to be tested. Finally, investigations into the underlying mechanisms of action and how individual stimulation parameters influence cellular behaviour and individual biological responses are ongoing in our laboratory; yet discussion of these results is beyond the scope of this article.

## Discussion

 In vitro direct ES devices can generally be classified into three main categories based on electrode configuration: (1) parallel-electrode systems (using pins [23], wires [38], or plates [28]), where electrodes are positioned in parallel on opposite sides of the cell culture area to apply an electric field across the cell or tissue cultures; (2) single working electrode systems, in which cells are cultured on one primary electrode located at the bottom of the culture chamber, while a second counter electrode completes the circuit through the medium, resulting in a vertical current relative to the cell layer; [68] and (3) multi-electrode or electrode-array systems, featuring multiple working electrodes integrated at the base of the culture area in various patterns [29]. In this work, we focus on a parallel-plate configuration, as it provides a uniform electric field that is perceived homogeneously by the majority of cells in the culture area [69]. This arrangement also enables repeated use of the same device without the need to replace electrodes.

**Fig. 7:**
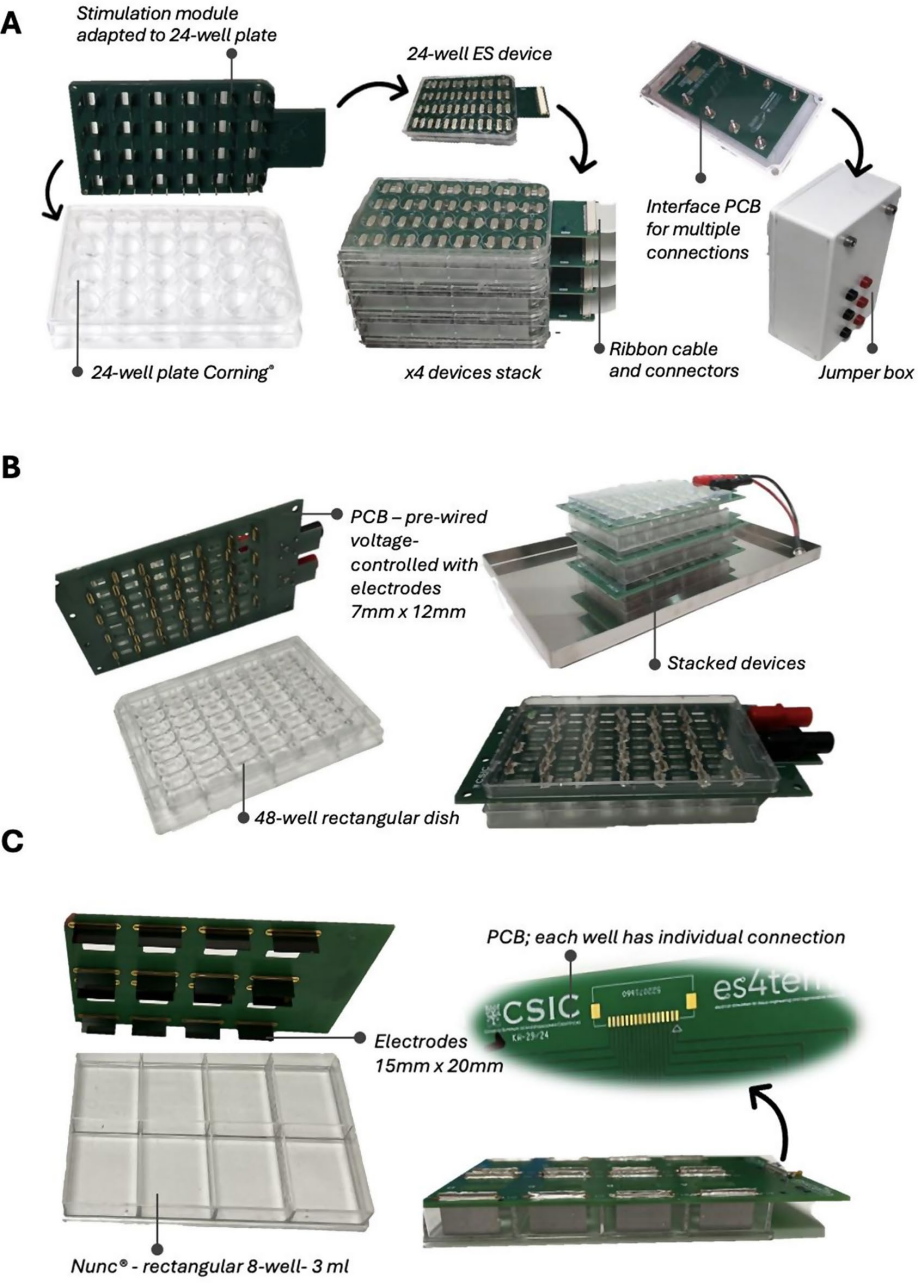
Examples of customized electrical stimulation (ES) devices. **(A)** ES device adapted for Corning^®^ 24-well plates, designed to deliver voltage-controlled stimulation with two distinct ES regimes and an integrated sham group within the same device. Assignment of stimulation and sham wells is configurable via an interface PCB. **(B)** ES device adapted for the 48-well myrPlate^®^ 3D cell/tissue culture platform, providing voltage-controlled stimulation; ES and sham groups are prewired in this configuration. **(C)** ES device adapted for a rectangular 8-well (3 mL) Nunc^®^ platform. Each electrode is individually wired to the ribbon connector, enabling extremely flexible operation in either voltage- or current-controlled modes via an interface PCB. Moreover, stimulation and sham group configurations are fully customizable. The fabrication files of all main and interface PCBs are available in supplementary Table S3

The platform introduced in this work is highly adaptable and compatible with a wide range of commonly used cell culture plat formats in various laboratories, Fig. 7. We have fabricated numerous devices adapted to 8-well micro-dishes (which are used for 2D culture and 3D organoid stimulation), Figs. 1 and 24-well plates Corning^®^ platform (which are used for retinal organoid stimulation), Figs. 7A and 48-well 3D cell/tissue culture myrPlate^®^ platform (which is used for heart organoid stimulation), Fig. 7B, and rectangular 8-well 3 ml Nunc^®^ platform (which is used for scaled 2D culture stimulation), Fig. 7C. PCB layouts and accessory choices are designed to be minimalistic, ensuring stackability and footprints comparable to standard culture dishes. Supplementary Table S3 provides ready-to-fabricate PCBs designed for various platforms tested in our lab. It is important to consider that the first step in developing an ES device is selecting an appropriate cell culture platform. This choice is critical for parallel electrode configurations because the distance between electrodes (d) directly determines the maximum electric field (E_e_) achievable within a safe applied voltage (V_e_) range according to *E*_*e*_ *= V*_*e*_*/d.* In addition, the platform geometry and culture area (volume) affect scalability, experimental design, and cost-efficiency. Key factors to consider are the nature of the biological question, the required number of replicates, sample size (n) and culture volume, as well as the cost of reagents and consumables. Since the ES device must be tailored to the culture platform, and post-fabrication modifications is limited, it is important to make a well-assessed decision early in the design process.

We have selected Pt as an optimum material for fabrication of electrodes in the method introduced in this work. Our choice has been made after deep evaluation of the reported bioelectrode materials, used both in vitro and in vivo. These materials include noble metals and alloys (e.g., Au [29, 30], Pt [37, 70, 71], Pt/Ir [72]), base metals and alloys (e.g., Ti [68], stainless steel [22]), conductive polymers (e.g., PEDOT: PSS [73, 74], SPAN [33]), and conductive oxides and carbon-based derivatives (e.g., ITO [66], graphene [75], bulk carbon [65, 76]). Supplementary Table S2 provides an overview of in vitro direct ES devices reported in literature, detailing electrode materials, configurations, and additional relevant discussions. While a wide range of materials has been explored, not all represent ideal choices. Some exhibit clear disadvantages, such as poor biocompatibility, limited conductivity, low CSC or mechanical instability, that are often overlooked. Ti is widely used in orthopedic and dental implants due to its biocompatibility, corrosion resistance, and favorable mechanical properties [77]. Many biology laboratories consider this biocompatible material as a proper electrode candidate for cell stimulation, due to its approved biocompatibility and being economic option compared to noble metals. However, Ti and its alloys immediately form a thin, self-passivating TiO₂ layer upon exposure to air [78]. While the oxide is biocompatible and corrosion-resistant, lack of the continuity of the layer allows erosion of Ti. These chemical and structural changes elevate electrochemical impedance and reduces CSC, diminishing its electrical performance as a bioelectrode [21, 36]. Cu, despite its high electrical conductivity and low-cost, undergoes rapid corrosion in biological fluids, releasing Cu⁺/Cu²⁺ ions that are cytotoxic at elevated concentrations, thus compromising both biocompatibility and long-term stability [79, 80]. Stainless steel alloys (e.g., SS316L, 316LVM, AISI316L) are inexpensive, mechanically durable, and moderately corrosion-resistant, which explains its common use in orthopedic and dental implants [81]. There are several reports on the use of this alloy family in ES devices [22, 32]. Nonetheless, stainless steel is not optimal choice for electrodes [25], as it forms a passive chromium oxide layer on its surface resulting in high impedance, low CSC compared to noble metals [45]. Another disadvantage is potential leaching of alloying elements (e.g., nickel, chromium), which may trigger inflammatory or cytotoxic responses during long-term use [82]. Carbon-based materials are biocompatible, suitable conductors and exhibit chemical inertness. However, carbon demonstrates various properties in different forms. As graphite, carbon exhibits compromised CSC and less chemical stability compared to noble metals [83, 84]. In the form of nanofibers or nanotubes, it shows enhanced electrochemical performance that, in some cases, surpasses that of noble metals [85, 86]. Conductive polymers such as PEDOT: PSS demonstrate significantly lower impedance and enhanced CSC compared with conventional metals, yet their long-term mechanical and electrochemical stability is still under evaluation [87]. The new generation of conductive polymers and carbon-based materials provide an attractive toolbox for developing in vitro platforms [88]. However, working with these materials requires advanced knowledge, specialized fabrication techniques, and established expertise, which may not be instantly available in every laboratory focused on biological research. Therefore, the choice of Pt is made due to excellent electrochemical performance, and commercial availability.

Electrochemical aspects of bioelectrical stimulation, particularly charge transfer mechanisms at the electrode–tissue interface, safe charge injection limits, and the distinction between reversible and irreversible electrochemical processes, have been extensively discussed in prior works [34, 45]. Merrill et al. established foundational principles linking stimulation waveforms, electrode materials, and electrochemical safety, while Boehler et al. later formalized standardized electrochemical characterization and reporting practices for neural-interface electrodes. Our approach in this paper is not separated from these established frameworks; rather, it operationalizes these electrochemical considerations during implementation. Specifically, we translate theoretical and experimental guidelines into reproducible equivalent circuit models and real-time monitoring circuits for controlled in vitro stimulation, thereby bridging electrochemical theory with system-level design and enabling dynamic supervision of stimulation conditions. This elevates prior conceptual guidelines into an implementable framework for robust stimulation control, prediction and consistency.

## Conclusions

We present a comprehensive framework for the development and operation of in vitro direct electrical stimulation (ES) devices, integrating two complementary aspects. First, we developed an economically feasible 8-well micro-dish ES device using parallel Pt electrodes. This device is readily scalable, and we have demonstrated extended configurations tailored to a variety of cell culture platforms, both for 2D monolayer cultures and 3D systems such as organoids and scaffold-based cultures. Second, we systematically characterized the electrochemical behaviour of the device and created an equivalent circuit model as a predictive tool. We established safe stimulation criteria for both voltage- and current-controlled methods and discussed the rational design of ES protocols. A proof-of-concept biological experiment using a voltage-controlled 8-well micro-dish device demonstrates the practical integration of these elements. Our results validated the reliability, reproducibility, and operational robustness of the framework. This work provides a standardized, open-access approach that unites device fabrication, electrochemical characterization, and functional ES implementation, establishing a versatile foundation for reproducible in vitro bioelectrical studies.

## Supplementary Information

Below is the link to the electronic supplementary material.

Supplementary Material 1

## Data Availability

Data is provided within the manuscript, supplementary files, and are available on open access [Zenodo](https://zenodo.org).

## Abbreviations

ES: Electrical stimulation
E: Electric field
d: Distance between electrodes
Q: Charge
Z: Impedance
Z_8−well_: Impedance correspond to 8 wells
C_dl_: Capacitance of the double layer
R_e_: Resistance of the culture media
R_f_: Faradaic or charge transfer resistance
τ: Time constant
V_source_: Voltage value introduced to the signal source
Z_output_: Impedance of the signal generator
I_source_: Current value introduced to the signal source
V_e_: Voltage inside the culture area
I_e_: Current inside the culture area
|E_e_|: Electric field inside the culture area
R_m_: Pre-set resistor
V_CH1_: Voltage value collected at channel 1
V_CH1_: Voltage value collected at channel 2
V_well_: Voltage applied to the well
Q_rec_: Charge recovered
Q_in_: Charge injected
BDNF: Brain derived neurotrophic factor
DMEM: Dulbecco’s modified eagle medium
FBS: Fetal bovine serum
PBS: Phosphate buffer saline
GlutaMAX: 200 mM L-alanyl-L-glutamine
LDH: Lactide de hydrogenase

## References

[1] Zhao S, Mehta AS, Zhao M. Biomedical applications of electrical stimulation. Cell Mol Life Sci. 2020;77:2681–99. 31974658 10.1007/s00018-019-03446-1 PMC7954539

[2] Haastert-Talini K, Grothe C. Electrical stimulation for promoting peripheral nerve regeneration. Int Rev Neurobiol. 2013;109:111–24. 24093609 10.1016/B978-0-12-420045-6.00005-5

[3] Tandon N, et al. Electrical stimulation systems for cardiac tissue engineering. Nat Protoc. 2009;4:155–73. 19180087 10.1038/nprot.2008.183 PMC2775058

[4] Becker R, Spadaro J. Electrical stimulation of partial limb regeneration in mammals. Bull New York Acad. 1972;48:627–41. PMC1806700 4503923

[5] Gan JC, Glazer PA. Electrical stimulation therapies for spinal fusions: current concepts. Eur Spine J. 2006;15:1301–1311. Preprint at: 10.1007/s00586-006-0087-y. 10.1007/s00586-006-0087-y PMC2438580 16604354

[6] Love MR, Palee S, Chattipakorn SC, Chattipakorn N. Effects of electrical stimulation on cell proliferation and apoptosis. J Cell Physiol. 2018;233:1860–76. 28452188 10.1002/jcp.25975

[7] Creasey GH et al. Clinical applications of electrical stimulation after spinal cord injury. J Spinal Cord Med. 2004;27:365–75. Preprint at: 10.1080/10790268.2004.11753774. 10.1080/10790268.2004.11753774 15484667

[8] Cheng H, Huang Y, Yue H, Fan Y. Electrical stimulation promotes stem cell neural differentiation in tissue engineering. Stem Cells Int. 2021;6697574. 10.1155/2021/6697574 PMC8081629 33968150

[9] Yuan X, Arkonac DE, Chao PHG, Vunjak-Novakovic G. Electrical stimulation enhances cell migration and integrative repair in the meniscus. Sci Rep. 2014;4. 10.1038/srep03674 PMC3891019 24419206

[10] Pettersen E, Shah FA, Ortiz-Catalan M. Enhancing osteoblast survival through pulsed electrical stimulation and implications for osseointegration. Sci Rep. 2021;11(123AD). 10.1038/s41598-021-01901-3 PMC8599699 34789829

[11] Preetam S et al. Electrical stimulation: a novel therapeutic strategy to heal biological wounds. 2024. 10.1039/d4ra04258a. 10.1039/d4ra04258a PMC11467653 39399261

[12] Leppik L, et al. Combining electrical stimulation and tissue engineering to treat large bone defects in a rat model. Sci Rep. 2018;8. 10.1038/s41598-018-24892-0 PMC5910383 29679025

[13] Balint R, Cassidy NJ, Cartmell SH. Electrical stimulation: a novel tool for tissue engineering. Tissue Eng Part B Rev. 2013;19:48–57. 22873689 10.1089/ten.TEB.2012.0183

[14] da Silva LP, Kundu SC, Reis RL, Correlo VM. Electric Phenomenon: A Disregarded Tool in Tissue Engineering and Regenerative Medicine. Trends Biotechnol. 2020;38:24–49. 31629549 10.1016/j.tibtech.2019.07.002

[15] Ben-Menachem E, Revesz D, Simon BJ, Silberstein S. Surgically implanted and non-invasive vagus nerve stimulation: a review of efficacy, safety and tolerability. Eur J Neurol. 2015;22:1260–8. 25614179 10.1111/ene.12629 PMC5024045

[16] Birmingham K, et al. Bioelectronic medicines: A research roadmap. Nat Rev Drug Discov. 2014;13:399–400. 24875080 10.1038/nrd4351

[17] Zhou J, Khateeb K, Yazdan-Shahmorad A. Early intervention with electrical stimulation reduces neural damage after stroke in non-human primates. Nat Commun. 2025;16:1–13. 40691150 10.1038/s41467-025-61948-y PMC12280128

[18] Kim H-S, Baby T, Lee J-H, Shin US, Kim H-W. Biomaterials-enabled electrical stimulation for tissue healing and regeneration. Med-X. 2024;2:1–27.

[19] Wang T, et al. Rehabilitation exercise–driven symbiotic electrical stimulation system accelerating bone regeneration. Sci Adv. 2024;10:6799. 10.1126/sciadv.adi6799 PMC10776020 38181077

[20] Sun J, Xie W, Wu Y, Li Z, Li Y. Accelerated Bone Healing via Electrical Stimulation. Adv Sci. 2025;12:2404190. 10.1002/advs.202404190 PMC12199592 39115981

[21] Zimmermann J, et al. Experimental and numerical methods to ensure comprehensible and replicable alternating current electrical stimulation experiments. Bioelectrochemistry. 2023;151:108395. 36773506 10.1016/j.bioelechem.2023.108395

[22] Silva JC, et al. Direct coupled electrical stimulation towards improved osteogenic differentiation of human mesenchymal stem/stromal cells: a comparative study of different protocols. Sci Rep. 2024;14:1–18. 38443455 10.1038/s41598-024-55234-y PMC10915174

[23] Wang Y, et al. Modulation of Osteogenesis in MC3T3-E1 Cells by Different Frequency Electrical Stimulation. PLoS ONE. 2016;11:e0154924. 27149625 10.1371/journal.pone.0154924 PMC4858221

[24] Leppik L, et al. Construction and use of an electrical stimulation chamber for enhancing osteogenic differentiation in mesenchymal stem/stromal cells in vitro. J Vis Exp. 2019;2019. 10.3791/59127 30774122

[25] Cibrão JR, et al. Development and application of a novel multi-channel in vitro electrical stimulator for cellular research. BMC Biomed Eng. 2025;7. 10.1186/s42490-025-00090-8 PMC11874659 40025549

[26] Cortes D, et al. BEaTS-α an open access 3D printed device for in vitro electromechanical stimulation of human induced pluripotent stem cells. Sci Rep. 2020;10:1–8. 32647145 10.1038/s41598-020-67169-1 PMC7347879

[27] Solazzo M, Monaghan MG. A Workflow to Produce a Low-Cost In Vitro Platform for the Electric-Field Pacing of Cellularised 3D Porous Scaffolds. ACS Biomater Sci Eng. 2023;9:4573–82. 37531298 10.1021/acsbiomaterials.3c00756 PMC10428090

[28] Pehlivanova V, et al. The role of alternating current electric field for cell adhesion on 2D and 3D biomimetic scaffolds based on polymer materials and adhesive proteins. J Mater Res. 2013;28:2180–6.

[29] Martín D, et al. DC electrical stimulation enhances proliferation and differentiation on N2a and MC3T3 cell lines. J Biol Eng. 2022;16:1–13. 36229846 10.1186/s13036-022-00306-8 PMC9563743

[30] Rabbani M, et al. A low-cost, scalable, and configurable multi-electrode system for electrical bio-interfacing with in-vitro cell cultures. Appl Sci 2024. 2023;14:162.

[31] Krueger S, et al. Establishment of a new device for electrical stimulation of non-degenerative cartilage cells in vitro. Int J Mol Sci 2021. 2021;22:394. 10.3390/ijms22010394 PMC7794805 33401406

[32] Visone R, Talò G, Lopa S, Rasponi M, Moretti M. Enhancing all-in-one bioreactors by combining interstitial perfusion, electrical stimulation, on-line monitoring and testing within a single chamber for cardiac constructs. Sci Rep. 2018;8:1–13. 30446711 10.1038/s41598-018-35019-w PMC6240103

[33] Min Y, et al. Sulfonated polyaniline-based organic electrodes for controlled electrical stimulation of human osteosarcoma cells. Biomacromolecules. 2013;14:1727–31. 23600698 10.1021/bm301221t

[34] Boehler C, Carli S, Fadiga L, Stieglitz T, Asplund M. Tutorial: guidelines for standardized performance tests for electrodes intended for neural interfaces and bioelectronics. Nat Protoc. 2020;15:3557–78. 33077918 10.1038/s41596-020-0389-2

[35] Kadan-Jamal K, et al. Electrical Stimulation of Cells: Drivers, Technology, and Effects. Chem Rev. 2025;125:6874–905. 40674565 10.1021/acs.chemrev.4c00468 PMC12355715

[36] Mobini S, et al. Effects of nanostructuration on the electrochemical performance of metallic bioelectrodes. Nanoscale. 2022;14:3179–90. 35142756 10.1039/d1nr06280h

[37] Mobini S, Leppik L, Barker JH. J. H. Direct current electrical stimulation chamber for treating cells in vitro. Biotechniques. 2016;60:95–8. 26842356 10.2144/000114382

[38] Mobini S, et al. In vitro effect of direct current electrical stimulation on rat mesenchymal stem cells. PeerJ. 2017;5:e2821. 28097053 10.7717/peerj.2821 PMC5237370

[39] Mobini S, González Sagardoy MU. Nanostructured electrodes for the electrical stimulation of cells in culture, devices, systems and procedures associates. ES Patent ES2887832 B2. 2021.

[40] Xue R, Mobini S, Cartmell S. A comparative study evaluating the ability of electro-bioreactors to deliver electric field for tissue engineering by finite element modelling. In: eCM Meeting TCES 99; London; 2016.

[41] Puglia MK, Bowen PK. Cyclic Voltammetry Study of Noble Metals and Their Alloys for Use in Implantable Electrodes. ACS Omega. 2022;7:34200–12. 36188288 10.1021/acsomega.2c03563 PMC9520554

[42] Zhou Y, Morris B, G. H., Nair M. Current and emerging strategies for biocompatible materials for implantable electronics. Cell Rep Phys Sci. 2024;5:101852.

[43] Tringides CM, Mooney DJ. Materials for Implantable Surface Electrode Arrays: Current Status and Future Directions. Adv Mater. 2022;34:2107207. 10.1002/adma.202107207 34716730

[44] Yang W, Gong Y, Li WA, Review. Electrode and Packaging Materials for Neurophysiology Recording Implants. Front Bioeng Biotechnol. 2021;8:622923. 33585422 10.3389/fbioe.2020.622923 PMC7873964

[45] Merrill DR, Bikson M, Jefferys JG. R. Electrical stimulation of excitable tissue: design of efficacious and safe protocols. J Neurosci Methods. 2005;141:171–98. 15661300 10.1016/j.jneumeth.2004.10.020

[46] Hudak EM, Mortimer JT, Martin H. B. Platinum for neural stimulation: voltammetry considerations. J Neural Eng. 2010;7:26005. 20208126 10.1088/1741-2560/7/2/026005

[47] Schuettler M. Electrochemical properties of platinum electrodes in Vitro: Comparison of six different surface qualities. Annual Int Conf IEEE Eng Med Biology - Proc. 2007;186–189. 10.1109/IEMBS.2007.4352254. 10.1109/IEMBS.2007.4352254 18001920

[48] Ohno H, et al. Fabrication of high-performance neural stimulation electrode through pulsed electrochemical deposition of IrOx onto nano-porous platinum. J Electrochem Soc. 2024;171:112505.

[49] Arias OA, Arias IOA. Artículo de Investigación on the thickness measurement of metallic thin films.

[50] Agustsson JS, et al. Electrical resistivity and morphology of ultra thin Pt films grown by dc magnetron sputtering on SiO2. J Phys Conf Ser. 2008;100:082006.

[51] García-Martín JM, Alvarez R, Romero-Gómez P, Cebollada A, Palmero A. Tilt angle control of nanocolumns grown by glancing angle sputtering at variable argon pressures. Appl Phys Lett. 2010;97.

[52] Garcia-Valenzuela A, et al. Voids and nanopores in nanocolumnar platinum thin films grown by magnetron sputtering and evaporation at oblique angles: A comparative analysis. Surf Interfaces. 2025;68:106667.

[53] Alvarez R, et al. Growth dynamics of nanocolumnar thin films deposited by magnetron sputtering at oblique angles. Nanotechnology. 2023;35:095705. 10.1088/1361-6528/ad113d 38035378

[54] Guette-Marquet S, Roques C, Bergel A. Theoretical analysis of the electrochemical systems used for the application of direct current/voltage stimuli on cell cultures. Bioelectrochemistry. 2021;139:107737. 33494030 10.1016/j.bioelechem.2020.107737

[55] Franks W, Schenker I, Schmutz P, Hierlemann A. Impedance characterization and modeling of electrodes for biomedical applications. IEEE Trans Biomed Eng. 2005;52:1295–302. 16041993 10.1109/TBME.2005.847523

[56] Srirussamee K, Mobini S, Cassidy NJ, Cartmell SH. Direct electrical stimulation enhances osteogenesis by inducing Bmp2 and Spp1 expressions from macrophages and preosteoblasts. Biotechnol Bioeng. 2019;116:3421–32. 31429922 10.1002/bit.27142 PMC6899728

[57] Srirussamee K, Xue R, Mobini S, Cassidy NJ, Cartmell SH. Changes in the extracellular microenvironment and osteogenic responses of mesenchymal stem/stromal cells induced by in vitro direct electrical stimulation. J Tissue Eng. 2021;12:204173142097414. 10.1177/2041731420974147 PMC7894594 33643602

[58] Bielfeldt M, et al. Discrimination between the effects of pulsed electrical stimulation and electrochemically conditioned medium on human osteoblasts. J Biol Eng. 2023;17:71. 37996914 10.1186/s13036-023-00393-1 PMC10668359

[59] Orazem ME, Tribollet B. Electrochemical impedance spectroscopy. Hoboken (NJ): John Wiley & Sons; 2008. 10.1002/9780470381588.

[60] Lazanas AC, Prodromidis MI. Electrochemical Impedance SpectroscopyA Tutorial. ACS Meas Sci Au. 2023;3:162–93. 37360038 10.1021/acsmeasuresciau.2c00070 PMC10288619

[61] Elgrishi N, et al. A Practical Beginner’s Guide to Cyclic Voltammetry. J Chem Educ. 2017;95:197–206.

[62] Hudak EM, Kumsa DW, Martin HB, Mortimer JT. Electron transfer processes occurring on platinum neural stimulating electrodes: calculated charge-storage capacities are inaccessible during applied stimulation. J Neural Eng. 2017;14:046012. 28345534 10.1088/1741-2552/aa6945 PMC5728108

[63] Wang X, et al. Pyruvate Protects Mitochondria from Oxidative Stress in Human Neuroblastoma SK-N-SH Cells. Brain Res. 2006;1132:1. 17174285 10.1016/j.brainres.2006.11.032 PMC1853247

[64] Khatib L, Golan DE, Cho M. Physiologic electrical stimulation provokes intracellular calcium increase mediated by phospholipase C activation in human osteoblasts. FASEB J. 2004;18:1903–5. 15385433 10.1096/fj.04-1814fje

[65] Karunasagara S, et al. Electrically-stimulated cellular and tissue events are coordinated through ion channel-mediated calcium influx and chromatin modifications across the cytosol-nucleus space. Biomaterials. 2025;314:122854. 39405824 10.1016/j.biomaterials.2024.122854

[66] Hlavac N, et al. Effects of Varied Stimulation Parameters on Adipose-Derived Stem Cell Response to Low-Level Electrical Fields. Ann Biomed Eng. 2021;49:3401–11. 34704163 10.1007/s10439-021-02875-z PMC10947800

[67] del Diego-Santiago M, et al. Bioelectric stimulation outperforms brain derived neurotrophic factor in promoting neuronal maturation. Sci Rep. 2025;15:(1):1–16. 10.1038/s41598-025-89330-4 PMC11807145 39922942

[68] Abasi S, Aggas JR, Venkatesh N, Vallavanatt IG, Guiseppi-Elie A. Design, fabrication and testing of an electrical cell stimulation and recording apparatus (ECSARA) for cells in electroculture. Biosens Bioelectron. 2020;147:111793. 31669804 10.1016/j.bios.2019.111793

[69] Zimmermann J, et al. Using a Digital Twin of an Electrical Stimulation Device to Monitor and Control the Electrical Stimulation of Cells in vitro. Front Bioeng Biotechnol. 2021;9:765516. 34957068 10.3389/fbioe.2021.765516 PMC8693021

[70] Diego-Santiago M, et al. Bioelectric stimulation outperforms BDNF in promoting neuronal maturations. Sci Rep; 2025. 10.1038/s41598-025-89330-4 PMC11807145 39922942

[71] Cogan SF. Neural stimulation and recording electrodes. Annu Rev Biomed Eng. 2008;10:275–309. 18429704 10.1146/annurev.bioeng.10.061807.160518

[72] Li L, et al. Electrochemical and biological performance of hierarchical platinum-iridium electrodes structured by a femtosecond laser. Microsyst Nanoeng. 2022;8:1–10. 36065436 10.1038/s41378-022-00433-8 PMC9440118

[73] Li J, et al. PEDOT:PSS-based bioelectronics for brain monitoring and modulation. Microsyst Nanoeng. 2025;11:(1):1–28. 10.1038/s41378-025-00948-w PMC12075682 40360495

[74] Kim S-M, et al. High-performance, polymer-based direct cellular interfaces for electrical stimulation and recording. NPG Asia Mater. 2018;10:255–65.

[75] Fabbri R, et al. Graphene oxide electrodes enable electrical stimulation of distinct calcium signalling in brain astrocytes. Nat Nanotechnol. 2024;19:1344–53. 38987650 10.1038/s41565-024-01711-4 PMC11405283

[76] Evers-Van Gogh IJA, et al. Electric pulse stimulation of myotubes as an in vitro exercise model: Cell-mediated and non-cell-mediated effects. Sci Rep. 2015;5:1–11. 10.1038/srep10944 PMC4473537 26091097

[77] Abd-Elaziem W, Darwish MA, Hamada A, Daoush WM. Titanium-Based alloys and composites for orthopedic implants Applications: A comprehensive review. Mater Des. 2024;241:112850.

[78] Pan J, Thierry D, Leygraf C. Electrochemical impedance spectroscopy study of the passive oxide film on titanium for implant application. Electrochim Acta. 1996;41:1143–53.

[79] Cortizo MC, De Mele MFL. Cytotoxicity of copper ions released from metal: Variation with the exposure period and concentration gradients. Biol Trace Elem Res. 2004;102:129–41. 15621934 10.1385/bter:102:1-3:129

[80] Liu Z, et al. Bivalent Copper Ions Promote Fibrillar Aggregation of KCTD1 and Induce Cytotoxicity. Sci Rep. 2016;6:1–8. 27596723 10.1038/srep32658 PMC5011690

[81] Barber CC, Burnham M, Ojameruaye O, McKee M. D. A systematic review of the use of titanium versus stainless steel implants for fracture fixation. OTA Int. 2021;4:e138. 34746670 10.1097/OI9.0000000000000138 PMC8568430

[82] Okazaki Y, Gotoh E. Metal release from stainless steel, Co–Cr–Mo–Ni–Fe and Ni–Ti alloys in vascular implants. Corros Sci. 2008;50:3429–38.

[83] O’Neill RD, Chang SC, Lowry JP, McNeil CJ. Comparisons of platinum, gold, palladium and glassy carbon as electrode materials in the design of biosensors for glutamate. Biosens Bioelectron. 2004;19:1521–8. 15093225 10.1016/j.bios.2003.12.004

[84] Brabec V, Schindlerová I. Electrochemical behaviour of proteins at graphite electrodes: Part III. The effect of protein adsorption. J Electroanal Chem Interfacial Electrochem. 1981;128:451–8.

[85] Sim S, et al. Neural probe integrated with low-impedance electrodes implemented using vertically aligned carbon nanotubes for three-dimensional mapping of neural signals. Sens Actuators B Chem. 2023;393:134124.

[86] Nimbalkar S, et al. Ultra-Capacitive Carbon Neural Probe Allows Simultaneous Long-Term Electrical Stimulations and High-Resolution Neurotransmitter Detection. Sci Rep. 2018;8:1–14. 29725133 10.1038/s41598-018-25198-x PMC5934383

[87] Teixeira HJ, Dias C, Veloso RC, Apolinário A, Ventura J. Tuning PEDOT:PSS low-impedance thin films with high charge injection for microelectrodes applications. Prog Org Coat. 2022;168:106894.

[88] Rocha I, Cerqueira G, Varella Penteado F. Córdoba de Torresi SI. Electrical Stimulation and Conductive Polymers as a Powerful Toolbox for Tailoring Cell Behaviour in vitro. Front Med Technol. 2021;3:670274. 35047926 10.3389/fmedt.2021.670274 PMC8757900

