# Supplementary material for: In vitro electrical stimulation devices: practical framework for design, fabrication, and operation
Source: J Biol Eng. 2026 Mar 20;20:76. doi: 10.1186/s13036-026-00664-7 (PMC13127091; doi:10.1186/s13036-026-00664-7)
Supplement: Supplementary file 1 — Supplementary Material 1 [file 13036_2026_664_MOESM1_ESM.docx]

***In Vitro* Electrical Stimulation Devices: Practical Framework for Design, Fabrication, and Operation**

*Gaurav Kulkarni, Jorge M. Garcia, Miriam Isasi Campillo, María Ujué González*, Sahba Mobini**

Instituto de Micro y Nanotecnología, IMN-CNM, CSIC (CEI UAM+CSIC), Isaac Newton 8, 28760 Tres Cantos, Madrid, Spain

*Corresponding Authors:

Sahba Mobini: [sahba.mobini@cisc.es](mailto:sahba.mobini@cisc.es);

María Ujué González: [maria-ujue.gonzalez@csic.es](mailto:maria-ujue.gonzalez@csic.es)

**Supplementary Materials**

**Contents:**

[**Table S1.** Cell culture platforms for incorporating direct-ES devices 2](#_Toc221895851)

[**Table S2.** Summary of in vitro direct electrical stimulation devices 3](#_Toc221895852)

[**Table S3.** Downloadable PCB design files 7](#_Toc221895853)

[**Table S4.** Suitable connectors and cables 9](#_Toc221895854)

[**Table S5.** Equivalent circuit models 10](#_Toc221895855)

[**Figure S6.** Behavior of voltage- and current-controlled systems 11](#_Toc221895856)

[**Table S7.** 3D-printable pieces 12](#_Toc221895857)

[**Figure S8.** Device in operation 12](#_Toc221895858)

[**Cost Estimation** 13](#_Toc221895859)

[**References** 14](#_Toc221895860)

**Table S1.** Cell culture platforms for incorporating direct-ES devices. Commercial culture platforms suitable for parallel electrodes configuration electrical stimulation (ES) devices, with the relevant associated parameters.

| **Platform/Commercial Brand** | **Shape/Surface area** | **~ Parallel plate electrode dimension** | **~ Distance between parallel electrodes** | **~ Maximum safe electric field achievable with 1 V applied voltage** |
| --- | --- | --- | --- | --- |
| 6-well plate/Several | Round; 9-9.6 cm^2^/well | 20 mm× 20 mm (estimated) | ~ 20-22 mm | ~50 mV/mm |
| 8-well plate/Cell culture plate Nunc® | Rectangular; 10-10.5 cm^2^/well | 15 mm × 20 mm (tested) | ~ 30-32 mm | ~30 mV/mm |
| 35 mm petri dish/Several | Round; 8-9 cm^2^/well | 20 mm× 20 mm(tested) | ~ 20-22 mm | ~50 mV/mm |
| 12-well plate/Several | Round; 3.5-3.9 cm^2^/well | 11 mm× 20 mm (estimated) | ~12-15 mm | ~ 80 mV/mm |
| 24-well plate/Corning®/Several | Round; 1.8-1.9 cm^2^/well | 8 mm × 21 mm(tested) | ~ 9-10 mm | ~ 100 mV/mm |
| 48-well 3D cell/tissue culture myrPlate® | Rectangular; stretcher | 7 mm × 11.5 mm(tested) | ~ 15-16 mm | ~ 65 mV/mm |
| 8-well micro dish/ibidi®; Sarstedt®; Millipore® | Rectangular; 0.7-0.8 cm^2^/well | 7 mm × 14 mm (tested) | ~ 8-10 mm | ~ 100 mV/mm |

**Table S2.** Summary of in vitro direct electrical stimulation devices. *In vitro* direct electrical stimulation devices with various electrode material, shape and configuration, reported in the literature.

| **Electrode Material** | **Electrode Shape** | **Electrode Configuration** | **ES Mode** | **Electric Field Distribution** | **Advantages** | **Disadvantages** | **Ref.** | **Commercial availability** |
| --- | --- | --- | --- | --- | --- | --- | --- | --- |
| **Platinum (Pt)** | | | | | | | | |
| Pure Pt | Rod/Pin Wire | Parallel inside the well/ 24-well plate | voltage-controlled | Not uniform | Simple Fabrication; Chemical/biological compatibility; Easy sterilization; Excellent electrochemical properties | Expensive; Not uniform field | 1 | No |
| Pure Pt | L-Shape Wire | Parallel inside the well/ 6-well plate | voltage-controlled | Not fully uniform | Simple Fabrication; Chemical/biological compatibility; Easy sterilization; Excellent electrochemical properties | Expensive; Local hotspots near tips; Protocol-specific | 2 | No |
| Pure Pt Coating | Rectangular Plate | Parallel Plate (Pt coated glass) on the sides of culture area/various | Flexible | Uniform | Simple fabrication; Cost-effective; Uniform current distribution; Chemical/biological compatibility; Easy sterilization; Excellent electrochemical properties | Fragile | Current Paper | No |
| Pt coated Ti | L-Shape | Parallel inside the well/ 12-well plate | Flexible | Not fully uniform | Chemical/biological compatibility; Inexpensive; Easy sterilization; Excellent electrochemical properties | Local hotspots near tips | 3 | No |
| Pt Coating | Coating Strip | Bottom of the 24 well plate | voltage-controlled | Not uniform | Perfusion integrated | Complex fabrication; Not uniform filed; Long-term stability | 4 | No |
| **Gold (Au)** | | | | | | | | |
| Pure Au Coating | Circular Dots | Array of Au electrodes imprinted in the bottom of 8-well micro dish/several | Flexible | Not uniform | Easy to use; Chemical/biological compatibility | One-time use; Expensive; Not uniform; Complex Fabrication | 56 | Applied BioPhysics |
| Pure Au Coating | Coated Strip | parallel PCB with the conductive tip (Au Coating) | Flexible | Not fully uniform | Chemical/biological compatibility; Easy sterilization | Not uniform; Local hotspots | 7 | No |
| **Titanium (Ti)** | | | | | | | | |
| Ti Alloy (Ti 6Al-4V Eli) | Circular Plate | Parallel plats on top and bottom - Direct culture on Ti Plate | Flexible | Not fully uniform | Integrated biosensing-stimulation platform; Chemical/biological compatibility | Poor electrochemical properties due to passivation layer; Complex fabrication | 8 | No |
| **Stainless Steel** | | | | | | | | |
| 316LVM | L-Shape | Parallel inside the well/ 6-well plate | Flexible | Not fully Uniform | Simple Fabrication; Inexpensive; Easy sterilization | Poor material stability issues; Local hotspots near tips | 9 | No |
| AISI 316L | Curved Plate | Parallel plats inside customized chamber | Flexible | Partially uniform | Modular integration; Perfusion Integrated | Complex fabrication/design; Poor electrode material choice | 10 | No |
| **Conductive polymers [ well reviewed by Ref. 18]** | | | | | | | | |
| Self-doped sulfonated polyaniline (SPAN) | Multiple lines | Embedded shape on the bottom of the cell culture chamber | Flexible | Not fully uniform | Scalable; Easy to use | Not compatible for 3D cultures; Distribution of current is not uniform for all culture area; one time use device | 11 | No |
| PolyPyrrol (PPY) | Multiple Rod | Multiple parallel rods inserted inside culture area in 3D shape | Flexible | Not uniform | Easy to use; 3D stimulation | long-term stability has not been studied; distribution of current has not studied | 12 | No |
| **Carbon** | | | | | | | | |
| Carbon | Flat Rectangular | Parallel Plate on the sides of culture area | Limited to the pre-programed functions of the ion-Optics | Uniform | Easy to use; Chemical/biological compatibility; | Electrodes might contain impurity and lack reproducibility; long-term stability issues; mechanically fragile | 13,14 | Ion Optics |
| Graphite | Flat Rectangular | Parallel Plate on the sides of culture area | Flexible | Uniform | Easy to use; Chemical/biological compatibility; Inexpensive | Electrodes might contain impurity and lack reproducibility; long-term stability issues; mechanically fragile | 15 | No |
| Carbon | Rod | Parallel rods on the bottom of P35 dishes | Flexible | Uniform | Easy to use; Chemical/biological compatibility; Inexpensive | Electrodes might contain impurity and lack reproducibility; long-term stability issues; Throughput limitation | 16 | No |
| Carbon | Rod | Parallel rods on the bottom of culture area | Flexible | Uniform | Easy to use; Chemical/biological compatibility; Modular; High throughput | Electrodes might contain impurity and lack reproducibility; long-term stability issues | 17 | No |

**Table S3.** Downloadable PCB design files. Gerber files (ready-to-fabricate) for stimulation, interface, and monitoring PCBs.

| **PCB description** | | **PCB Layout** | **Design and Gerber files** |
| --- | --- | --- | --- |
| 1. Device PCB for 8-well micro dish; Voltage-controlled pre-connected | | 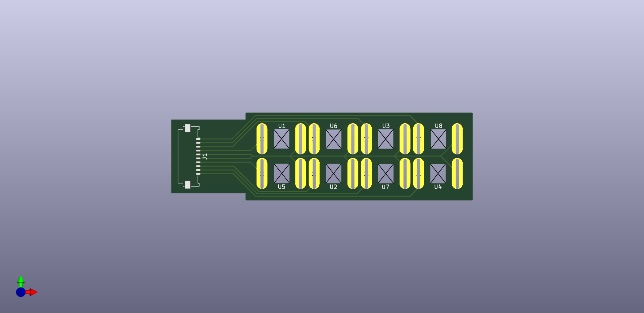 | [Link S3.1](https://doi.org/10.5281/zenodo.16993416) |
| 1. Device PCB for 8-well micro dish; Current-controlled pre-connected | | 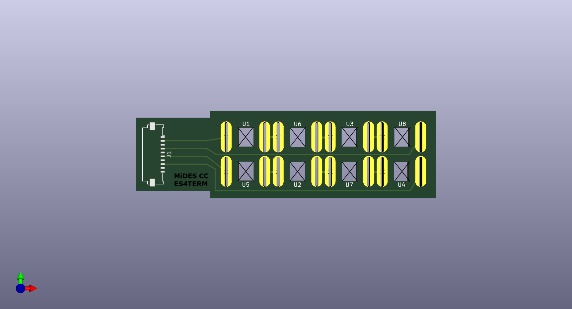 | [Link S3.2](https://doi.org/10.5281/zenodo.16992725) |
| 1. Interface PCB for 8-well micro dish; Voltage-controlled | SHAM/ES divider for 4 wells | 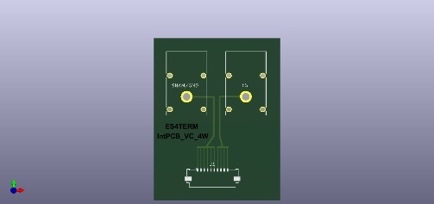 | [Link S3.3.1](https://doi.org/10.5281/zenodo.16993454) |
|  | All ES for 8 wells | 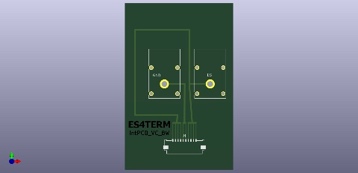 | [Link S3.3.2](https://doi.org/10.5281/zenodo.16993041) |
| 1. Interface PCB for 8-well micro dish; Current-controlled | Flexible for connecting 4 well and 8 well | 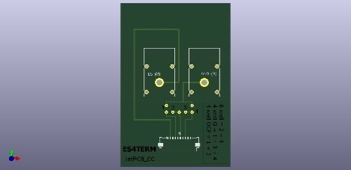 | [Link S3.4](https://doi.org/10.5281/zenodo.16993204) |
| 1. Device PCB for NUNC® 8-well –individual input- adaptable to voltage and current | | 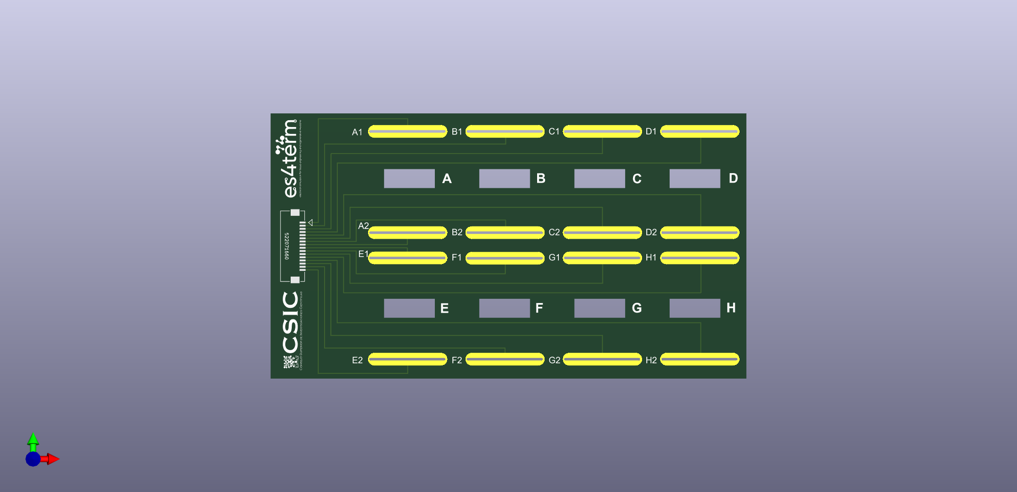 | [Link S3.5](https://doi.org/10.5281/zenodo.16992753) |
| 1. Voltage- controlled interface for NUNC® 8-well device | | 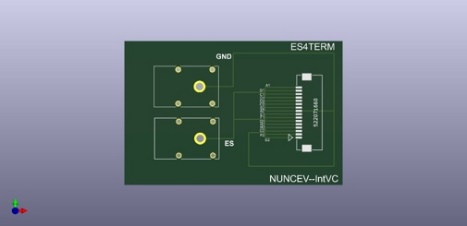 | [Link S3.6](https://doi.org/10.5281/zenodo.16992952) |
| 1. Current- controlled interface for NUNC® 8-well device | | 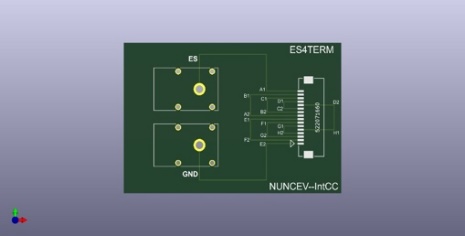 | [Link S3.7](https://doi.org/10.5281/zenodo.16992974) |
| 1. Device PCB for myrPlate® 48-well pre-wired for voltage-controlled - customized for half stimulation and half sham | | 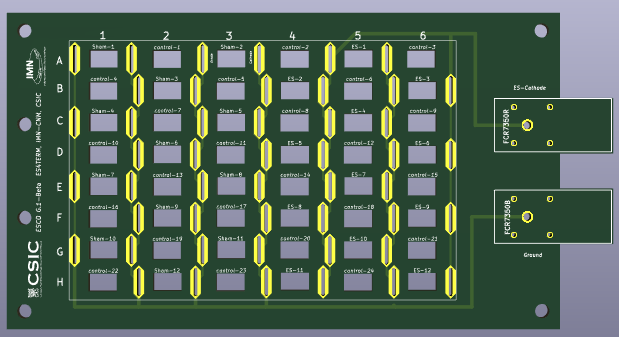 | [Link S3.8](https://doi.org/10.5281/zenodo.16993506) |
| 1. Device PCB for 24-well Corning® Plate - All wells with individual input | | 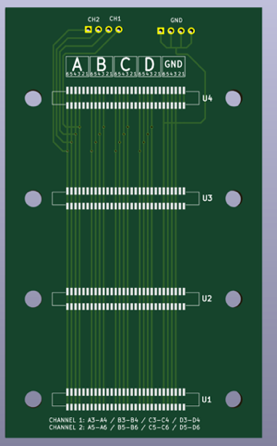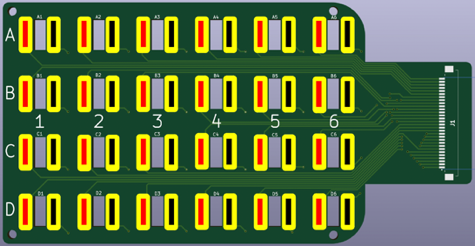 | [Link S3.9](https://doi.org/10.5281/zenodo.17406249) |
| 1. Interface PCB for 24-well plate for grouping and assigning to different ES and sham in voltage-controlled mode-for 4 devices in parallel | |  | [Link S3.10](https://doi.org/10.5281/zenodo.17406149) |
| 1. Monitoring PCB | | 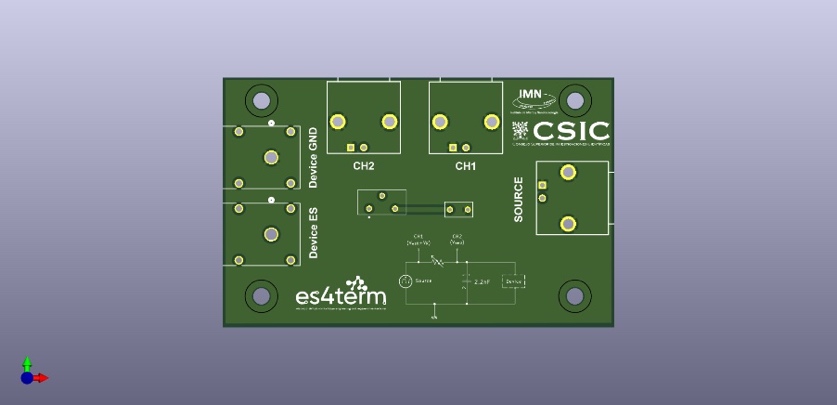 | [Link S3.11](https://doi.org/10.5281/zenodo.16993166) |

| **Item and function description** | | | **Commercial “RS” CAT No.** | **Device** |
| --- | --- | --- | --- | --- |
| Device PCB –Interface Connection | FPC connector  10-way | 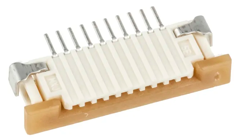 | 514-4329 | 8-well micro dish |
|  | FPC cable  10-way | 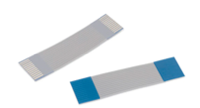 | 163-1520 | 8-well micro dish |
| Interface- Source Connection | Banana Connector | 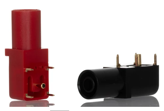 | 187-3802 | All |
|  | Banana Cable | 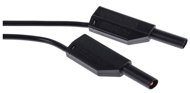 | 404-351 | All |
| Device PCB – Interface Connection | FPC Connector 16-way | 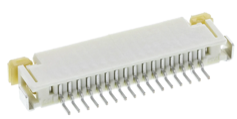 | 670-6760 | 8-well Nunc |
|  | FPC Cable  16-way | 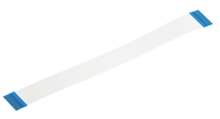 | 458-9210 | 8-well Nunc |
| BNC Connector | | 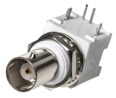 | 700-9411 | Measuring Circuit |
| Trimmer pot- Adjustable resistor 1KΩ Max | | 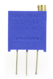 | 522-0063 | Measuring Circuit |
| Capacitor, 2.2 nF | | 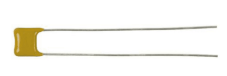 | 699-4885 | Measuring Circuit |

**Table S4.** Suitable connectors and cables. List of connectors and cables compatible with various platforms.

**Table S5.** Equivalent circuit models, downloadable files.

| **Device** | **Equivalent Circuit Model (Example)** | **Link** |
| --- | --- | --- |
| 1. Device with Pt TF electrodes – Voltage- and current -controlled (several configurations included) | 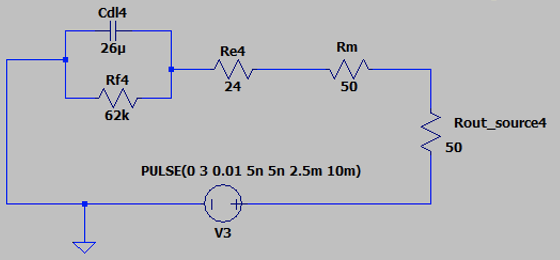8-well micro-dish device - monitoring circuit included - voltage controlled - monophasic pulse | [Link S5.1](https://doi.org/10.5281/zenodo.17011703) |
| 2. Device with Pt NC electrodes– Voltage-controlled (several configurations included) | 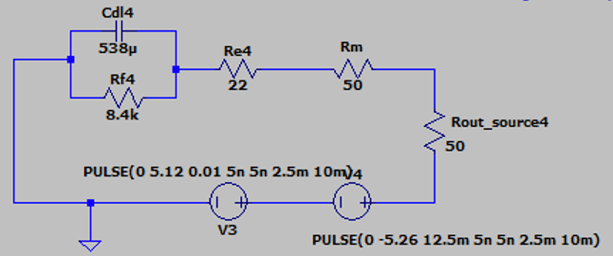8-well micro-dish device - monitoring circuit included - voltage controlled - biphasic pulse | [Link S5.2](https://doi.org/10.5281/zenodo.17011520) |

**
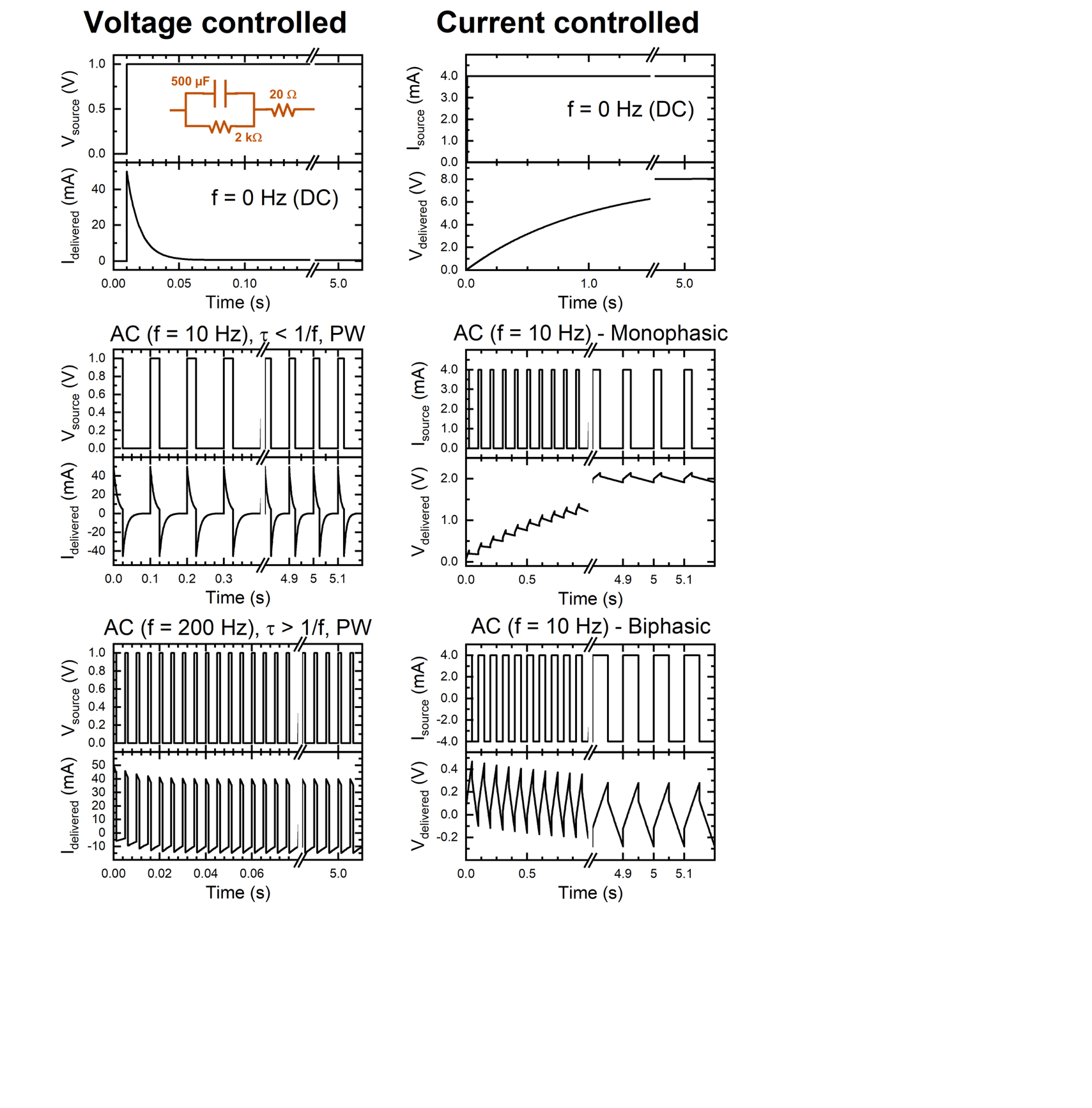
****Figure S6.** Behavior of voltage- and current-controlled systems. The equivalent circuit corresponding to a direct electrical stimulation (ES) device based on parallel electrode configuration has been used. The electric circuit, together with the specific values used in the calculations, is shown in the figure, upper part: it consists of a capacitor (500 µF) in parallel with a resistor (2 kΩ), both in series with a second resistor (20 Ω). The time constant, τ, of this circuit is 10 ms. In the case of voltage-controlled circuit (left), the evolution with time of the current delivered through the 20 Ω resistor, I_delivered_, is plotted for: continuous stimulation, f = 0 Hz (top panel); monophasic pulses with period (1/f) and pulse width (PW) bigger than the time constant (middle panel); and monophasic pulses with period and PW smaller than τ (bottom panel). The decay of I_delivered_, can be observed. In the case of current-controlled circuit (right), the temporal evolution of the voltage delivered between both electrodes, V_delivered_, is plotted for: continuous stimulation (top panel); monophasic pulses (middle panel); and biphasic symmetric pulses (bottom panel). The build-up of V_delivered_ can be seen.

**Table S7.** 3D-printable pieces.

| **3D-Printable Item** | **Link to 3D *.STL design file** |
| --- | --- |
| 1. Rack for interface PCB | [Link S7.1](https://doi.org/10.5281/zenodo.17013397) |
| 1. Rack for measuring PCB | [Link S7.2](https://doi.org/10.5281/zenodo.17406954) |
| 1. Lid for 8-well micro dish | [Link S7.3](https://doi.org/10.5281/zenodo.17235832) |

**Figure S8.** Device in operation. Schematic representation of the devices in operation inside the incubator showing connections to the signal source, monitoring circuit, and computer interface. Option 1: device connected to the measuring circuit and oscilloscope; Option 2: device connected to the measuring circuit without measuring; Option 3: Device directly connected to the source, which is the traditional way of stimulation, but limits the adjustment and control.

A short video demonstrating the device in operation is provided at the following link: [[Link to Video](https://doi.org/10.5281/zenodo.18802432)].


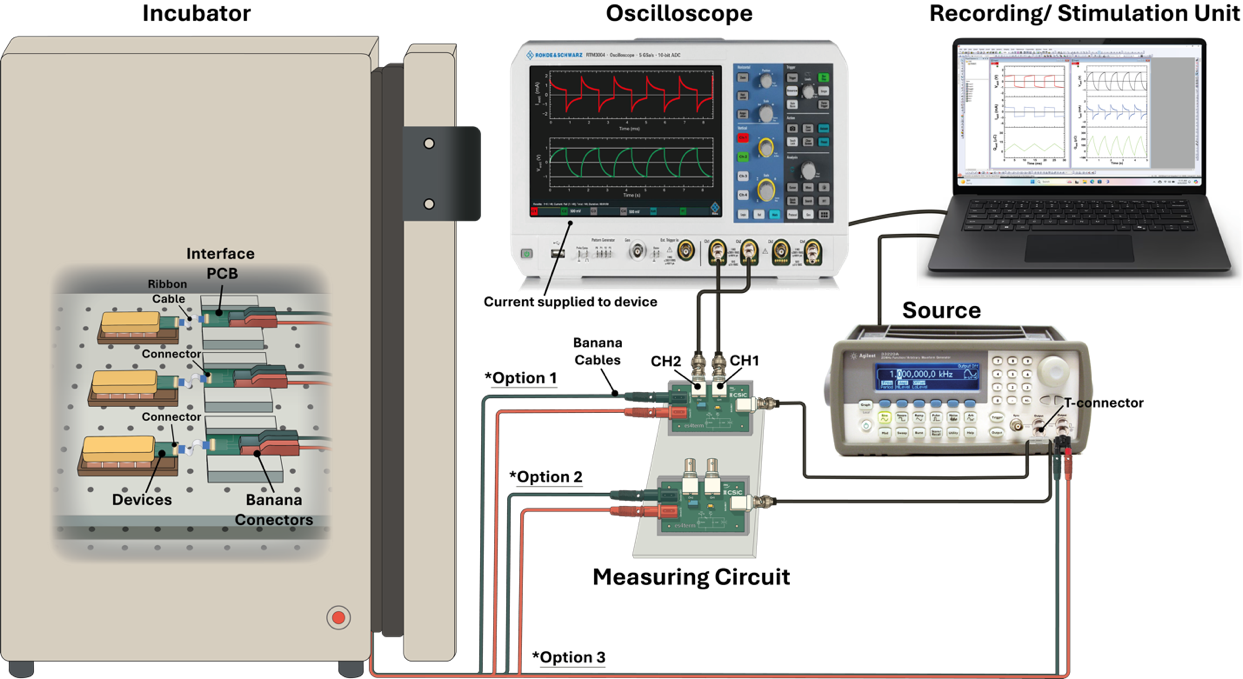


# **Cost Estimation**

| **Item/Service*** | **~Price/Unit ($)** | **Unit** | **Total Price** |
| --- | --- | --- | --- |
| Main PCB** | 10.00 € | 1 | 10.00 € |
| Interphase PCB | 7.00 € | 1 | 7.00 € |
| Measurement PCB | 20.00 € | 1 | 20.00 € |
| Electrodes- Substrates | 2.75 € | 16 | 44.00 € |
| Electrodes- Evaporation /Hour*** | 100.00 € | 6 | 600.00 € |
| Connectors | 15.00 € | 2 | 30.00 € |
| Ribbon cable | 4.00 € | 1 | 4.00 € |
| **Estimated Total Price:** | | | **715.00 €** |

** The estimation is done for developing one device adapted to 8-well micro-dish. All prices are estimated based on information collected between year 2020 and 2025.*

*** The PCB fabrication price estimated from a private company based in Spain.*

**** The evaporation service price is estimated averaging private and public research facilities fees in Europe.*

# **References**

1. Wang, Y. *et al.* Modulation of Osteogenesis in MC3T3-E1 Cells by Different Frequency Electrical Stimulation. *PLoS One* **11**, e0154924 (2016).

2. Srirussamee, K., Mobini, S., Cassidy, N. J. & Cartmell, S. H. Direct electrical stimulation enhances osteogenesis by inducing Bmp2 and Spp1 expressions from macrophages and preosteoblasts. *Biotechnol Bioeng* **116**, 3421–3432 (2019).

3. Cibrão, J. R. *et al.* Development and application of a novel multi-channel in vitro electrical stimulator for cellular research. *BMC Biomed Eng* **7**, (2025).

4. Blume, G., Müller-Wichards, W., Goepfert, C., Pörtner, R. & Müller, J. Electrical Stimulation of NIH-3T3 Cells with Platinum-PEDOT-Electrodes Integrated in a Bioreactor. *Open Biomed Eng J* **7**, 125–132 (2013).

5. Martín, D., Ruano, D., Yúfera, A. & Daza, P. Electrical pulse stimulation parameters modulate N2a neuronal differentiation. *Cell Death Discov* **10**, 1–9 (2024).

6. Martín, D. *et al.* DC electrical stimulation enhances proliferation and differentiation on N2a and MC3T3 cell lines. *J Biol Eng* **16**, 1–13 (2022).

7. Rabbani, M. ; *et al.* A Low-Cost, Scalable, and Configurable Multi-Electrode System for Electrical Bio-Interfacing with In-Vitro Cell Cultures. *Applied Sciences 2024, Vol. 14, Page 162* **14**, 162 (2023).

8. Abasi, S., Aggas, J. R., Venkatesh, N., Vallavanatt, I. G. & Guiseppi-Elie, A. Design, fabrication and testing of an electrical cell stimulation and recording apparatus (ECSARA) for cells in electroculture. *Biosens Bioelectron* **147**, 111793 (2020).

9. Silva, J. C. *et al.* Direct coupled electrical stimulation towards improved osteogenic differentiation of human mesenchymal stem/stromal cells: a comparative study of different protocols. *Sci Rep* **14**, 1–18 (2024).

10. Visone, R., Talò, G., Lopa, S., Rasponi, M. & Moretti, M. Enhancing all-in-one bioreactors by combining interstitial perfusion, electrical stimulation, on-line monitoring and testing within a single chamber for cardiac constructs. *Sci Rep* **8**, 1–13 (2018).

11. Min, Y. *et al.* Sulfonated polyaniline-based organic electrodes for controlled electrical stimulation of human osteosarcoma cells. *Biomacromolecules* **14**, 1727–1731 (2013).

12. Zhang, Q. *et al.* Electrical Stimulation with a Conductive Polymer Promotes Neurite Outgrowth and Synaptogenesis in Primary Cortical Neurons. *Sci Rep* **8**, 1–10 (2018).

13. Cortes, D. *et al.* BEaTS-α an open access 3D printed device for in vitro electromechanical stimulation of human induced pluripotent stem cells. *Sci Rep* **10**, 1–8 (2020).

14. Staehlke, S. *et al.* Pulsed Electrical Stimulation Affects Osteoblast Adhesion and Calcium Ion Signaling. *Cells 2022, Vol. 11, Page 2650* **11**, 2650 (2022).

15. Pehlivanova, V. *et al.* The role of alternating current electric field for cell adhesion on 2D and 3D biomimetic scaffolds based on polymer materials and adhesive proteins. *J Mater Res* **28**, 2180–2186 (2013).

16. Gabetti, S. *et al.* Versatile electrical stimulator for cardiac tissue engineering—Investigation of charge-balanced monophasic and biphasic electrical stimulations. *Front Bioeng Biotechnol* **10**, 1031183 (2023).

17. Solazzo, M. & Monaghan, M. G. A Workflow to Produce a Low-Cost In Vitro Platform for the Electric-Field Pacing of Cellularised 3D Porous Scaffolds. *ACS Biomater Sci Eng* **9**, 4573–4582 (2023).

18. Rocha, I., Cerqueira, G., Varella Penteado, F. & Córdoba de Torresi, S. I. Electrical Stimulation and Conductive Polymers as a Powerful Toolbox for Tailoring Cell Behaviour in vitro. *Front. Med. Technol.* **3**, 670274 (2021).
